# A new amidohydrolase and β-oxidation–like pathway for piperine catabolism in soil actinomycetes

**DOI:** 10.1016/j.jbc.2025.110908

**Published:** 2025-11-05

**Authors:** Pu Jian, Takuto Kumano, Mio Kimura, Makoto Kurisaki, Yoshiteru Hashimoto, Michihiko Kobayashi

**Affiliations:** 1 Graduate School of Life and Environmental Sciences, University of Tsukuba, Ibaraki, Japan; 2 Microbiology Research Center for Sustainability, University of Tsukuba, Ibaraki, Japan; 3 Tsukuba Institute for Advanced Research (TIAR), University of Tsukuba, Ibaraki, Japan; 4 Center for Quantum and Information Life Sciences, University of Tsukuba, Ibaraki, Japan

**Keywords:** black pepper, alkaloid, bacteria, *Rhodococcus*

## Abstract

Understanding the microbial degradation of plant-derived specialized metabolites is important for gaining insight into plant microbial diseases. Piperine is a unique alkaloid found in peppers and plays a protective role essential for pepper tree growth. However, how environmental microbiomes interact with pepper trees, especially *via* the interaction with piperine, has remained completely unknown. To elucidate microbial piperine metabolism, we previously screened *Rhodococcus ruber* No. 14, an actinomycete that catabolizes piperine from a surface soil sample containing detritus. In this actinomycete, here, we discovered a piperine hydrolase (named PipM) that catalyzes the hydrolysis of the tertiary amide bond in piperine. While no enzyme had been reported to hydrolyze the piperine-type tertiary amide bond, PipM showed amino acid sequence similarity to amidohydrolase family enzymes and, like them, required metal ions for its activity. The mRNA-Seq and genome analysis revealed a gene cluster of 48 genes, among which 42 genes, including *pipM,* were upregulated by adding piperine into the culture medium. Through gene expression and biochemical analysis, four genes (named *pipU, V, W, and X*) were identified to encode enzymes that catalyze the β-oxidation-like reaction to convert piperic acid to piperonylic acid. Our study unraveled microbial piperine metabolism and would provide insight into interactions between pepper trees and environmental microbiomes.

Alkaloids are a broad class of naturally occurring, nitrogen-containing compounds with diverse bioactive properties, and the existence of plant- or microbe-derived alkaloids can profoundly influence microbial communities in natural environments (1, 2).

Piperine is a plant-derived alkaloid which was initially isolated from *Piper nigrum* (black pepper) and can also be produced by other members of the family Piperaceae (3), which could mainly be seen in semi-tropical or tropical areas. Piperine exhibits various bioactive effects that may be important for the growth of these plants (4). Piperine contains a tertiary amide bond and a characteristic methylenedioxyphenyl group (Fig. 1*A*). Most tertiary amides of alkaloids form cyclic structures (*e.g.*, caffeine and febrifugine; Fig. 1*B*), and there are some reported cases about how microorganisms metabolize alkaloids with such structures (5). In contrast, piperine contains an atypical tertiary amide (piperine-type tertiary amide) bond that is between its heterocyclic nitrogen and the carbonyl group outside the ring, forming an acyclic structure which is distinctively different from other known alkaloids (Fig. 1, *A* and *B*). As far as we know, microbial metabolism of alkaloids that include acyclic tertiary amide structures like piperine has not been previously reported. Moreover, pepper trees, the source of an important seasoning, are now cultured worldwide (6). Considering that piperine occupies 4% to 9% dry weight of black pepper fruit (7) and its antimicrobial activity, piperine may play a defensive role by interfering the local microbial communities *via* the fruits that fell to the ground. However, microbes can seemingly circumvent the antimicrobial properties of piperine; the mechanism by which they disarm the piperine defense through catabolism remains unknown.

**Figure 1:**
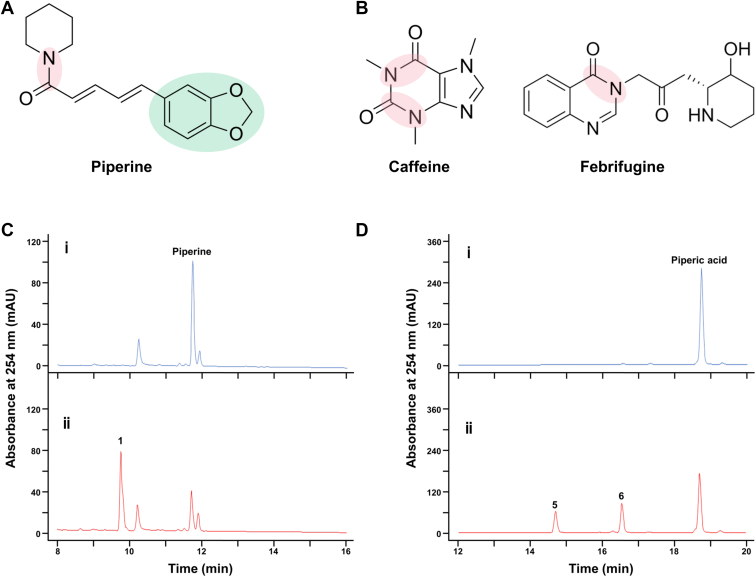
**Products of piperine metabolism.***A*, chemical structure of piperine shows one tertiary amide bond (*shaded pink*) and one methylenedioxyphenyl group (*shaded green*). The tertiary amide bond is outside of the hetero carbon-nitrogen ring, forming a characteristic acyclic tertiary amide. *B*, chemical structures of representative alkaloids that contain cyclic tertiary amide bond (*left*, caffeine; *right*, febrifugine), in which the tertiary amide bonds (*shaded pink*) are located inside the hetero carbon-nitrogen rings. *C*, LC-MS analysis of reaction mixtures of piperine and *R. ruber* No. 14 culture supernatants. HPLC chromatograms of reaction mixtures at (i) 0 min and (ii) after incubation for 3 h. The degradation product of piperine is indicated as **1**. The decrease of piperine and the formation of two products **1** was detected by LC-MS analysis after 3 h reaction between piperine and *R. ruber* No. 14 culture supernatants. *D*, LC-MS analysis of reaction mixtures of piperic acid and PipU, V, W and X HPLC chromatograms of reaction mixtures at (i) 0 min and (ii) after incubation for 4 h. The two products are indicated as **5** and **6**. The decrease of piperic acid and the formation of two products were detected by LC-MS analysis after 4 h reaction between piperic acid and purified PipU, V, W and X.

Therefore, elucidating microbial piperine metabolism would provide breakthroughs to novel enzymatic mechanisms as well as the theoretical fundamentals to understand how soil microorganisms interact with such important commercial crops.

Soil microorganisms with specialized metabolic abilities can usually be found in conditions where certain plant-derived metabolites exist in nature (8, 9). Previously, we isolated *Rhodococcus ruber* No. 14, an actinomycete that is capable in catabolizing piperine as a sole nitrogen source from the detritus soil sample collected Okinawa (10).

Actinomycetes are a diverse group of gram-positive G/C-rich bacteria that are widely distributed across various ecosystems (11). The natural products research community focuses on characterizing the diverse secondary metabolites produced by actinomycetes (12, 13); however, these organisms can also degrade multiple environmental pollutants, a property that warrants additional investigation (14, 15). *Rhodococcus*, a genus of actinomycetes that has significant biodegradation potential, have been reported to break down aromatic compounds, steroids, and even lignin (16, 17). As far as we can ascertain, the microbial metabolism of piperine and related enzymes have never been previously found. Given the diverse and unique degradative capabilities of *Rhodococcus*, we anticipated the discovery of new piperine metabolic pathways and associated enzymes from *R. ruber* No. 14.

From strain No.14, we found PipA, an enzyme that decomposes the methylenedioxyphenyl group of piperonylic acid (10). However, how piperine is metabolized in this bacterium is unknown. Here, we report the discovery of piperine hydrolase (PipM) which catalyzes the initial breakdown of piperine, as well as other four enzymes that function in the following steps of piperine metabolism. Database analysis indicated that PipM belongs to amidohydrolase superfamily (AHS), a diverse group of metal-depending enzymes. This study elucidated how soil microorganism utilizes this plant-derived alkaloid, unveiled the metabolic pathway of piperine and revealed the key enzymes that function in this pathway.

## Results

### Determination of piperine metabolite structure

**Figure 2:**
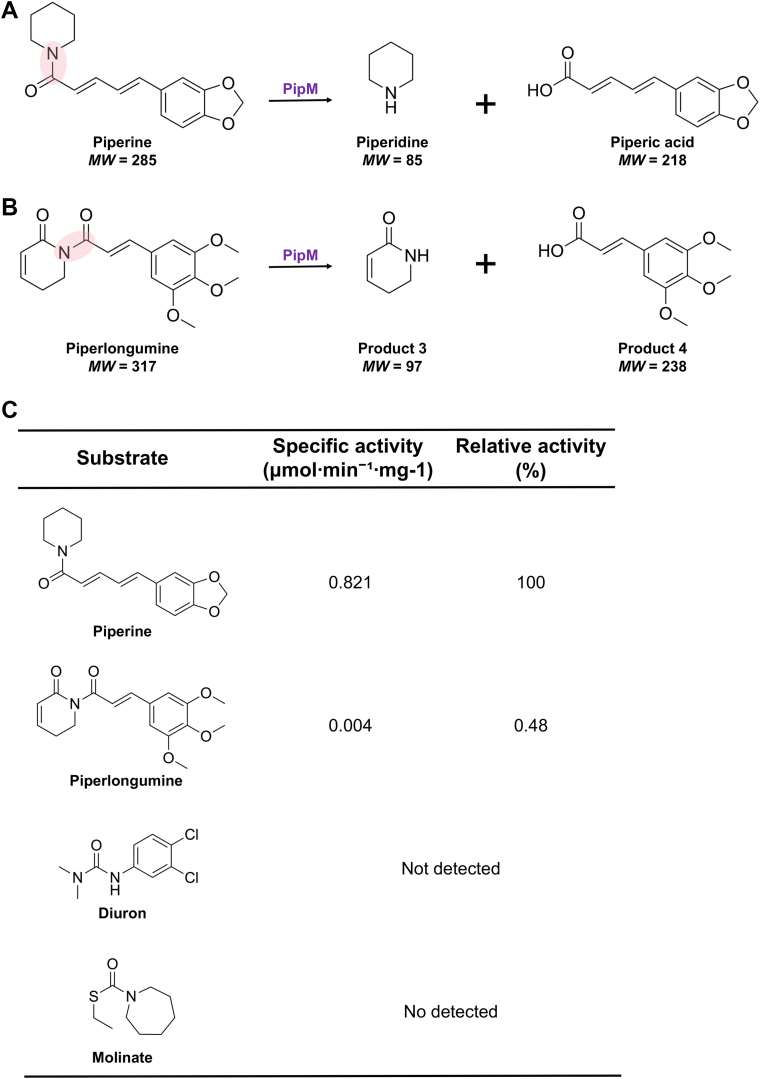
**Substratespecificity of PipM.** Reactions of PipM with (*A*) piperine and (*B*) piperlongumine. The hydrolyzed tertiary amide bonds of substrates are highlighted in *shaded pink*. *C*, activities of PipM towards candidate substrates.

Some soil actinomycetes exert primary biodegradative activity by secreting extracellular enzymes into the surrounding environment (18). After incubating piperine with *R. ruber* No. 14 culture supernatants for 3 h *in vitro,* liquid chromatography-mass spectrometry (LC-MS) found significantly less piperine and an unidentified product (Product **1**, Fig. 1*C*) with a molecular mass of 218 (Fig. S1*C*). Reactions that were stopped at 0 min were used as controls for comparison. This was consistent with the calculated mass of piperic acid, which might be a potential metabolic intermediate of piperine. The LC-MS analysis revealed that the retention time and UV spectrum of Product **1** were the same as those of authentic piperic acid (Fig. S1, *A* and *B*). These findings indicated product **1** to be piperic acid. The formation of piperic acid indicated the piperine hydrolysis under the addition of *R. ruber* No. 14 culture supernatants (Fig. 2). Furthermore, after derivatizing the amines in the reaction solution with NBD-F, NBD-F derivative of piperidine was detected by LC-MS/MS analysis (Fig. S2).

### Identification, heterologous expression, and purification of PipM

We isolated an enzyme in *R. ruber* No. 14 culture supernatants that degraded piperine using four types of chromatography, and SDS-PAGE revealed a candidate protein band corresponding to the enzyme (Fig. S3, *A* and *B*). We transferred the band to a PVDF membrane and determined that the *N*-terminal amino acid sequence of this purified protein was NSFALSNV. A local BLAST search identified an open reading frame (ORF) consisting of 1389 nucleotides with a deduced amino acid sequence that corresponded to the *N*-terminal sequence. We named this ORF *pipM*, and BLAST analysis classified the deduced protein as an amidohydrolase (Fig. S4). Also, the analysis using SignalP 6.0 indicated that there are no signal peptide in the deduced amino acid sequence of *pipM*. To further investigate the detailed properties of this enzyme, *pipM* was cloned and the recombinant enzyme was expressed and purified (Table S1, Fig. S3, *C* and *D*).

The results of gel-filtration column chromatography showed that the molecular mass of native PipM was ∼ 220 kDa (Fig. S5). Considering that the calculated molecular mass of the PipM monomer was ∼ 50 kDa, which agreed with the SDS-PAGE results (Fig. S3*A*), we assumed that native PipM would be a homotetramer under natural conditions.

### Biochemical properties of PipM

We assessed the stability and dependency of PipM within the temperature of 10 °C to 80 °C and found that 25% of the enzyme activity was retained at 60 °C and abolished at > 70 °C. The optimal temperature was ∼ 50 °C. (Fig. S6, *A* and *B*). Examinations of the stability and dependency of PipM with the pH ranges of 2.0 to 12.0 revealed that the reaction was optimal at pH ∼ 7.0, and the enzyme activity was maintained by incubating between pH 4.0 to 10.0. (Fig. S6, *C* and *D*).

We analyzed the kinetic parameters of pipM under 0.001 to 0.5 mM piperine. The specific activity at each substrate concentration was determined within the range where product formation increased linearly, and substrate consumption was found to be less than 10% in all reactions. Nonlinear regression using the Michaelis-Menten equation and Lineweaver-Burk plots showed that PipM had *K*_m_ = 2.11 ± 0.31 μM; *V*_max_ = 0.313 ± 0.009 (μmol·min^−1^ mg^−1^); and *k*_cat_ = 0.26 s^−1^ (Fig. S7).

We analyzed the metal-binding properties of PipM and the inhibitory effects of small molecules on PipM. We found that PipM contained Zn^2+^ (Table S3), and it was inhibited by chelating and serine-modifying agents (Fig. S8).

We selected piperine, piperlongumine, diuron (3-(3,4-dichlorophenyl)-1,1-dimethylurea), and molinate (*S*-ethyl azepane-1-carbothioate) as candidate compounds to determine the substrate specificity of PipM. Like piperine, piperlongumine is an alkaloid synthesized by *P. nigrum* that also has a tertiary amide bond (19). Diuron and molinate are artificial herbicides that can be decomposed by PipM homologues, PuhB and MolA (20, 21). PuhB has been isolated from *Mycobacterium brisbanense* strain JK1, which is involved in diuron degradation (20). MolA, on the other hand, was identified in *Gulosibacter molinativorax* ON4^T^, which catalyzes molinate hydrolysis (21). Sequence alignment showed that PuhB and MolA share 65% and 64% similarity with PipM, respectively. Based on the above, we further investigated piperlongumine, diuron, and molinate. The LC-MS analysis revealed no significant changes in diuron or molinate, but a decline in piperlongumine after incubation with PipM (Figs. S9–S11). The LC-MS analysis also identified two reaction products with molecular masses of 238 and 97 that formed while piperlongumine decreased (Fig. S9). These results indicated that PipM had hydrolyzed the tertiary amide bond of piperlongumine (Fig. 2*B*). Further investigation revealed a slow reaction rate between PipM and piperlongumine, with a relative activity of only 0.48% compared with that of piperine (Fig. 2*C*).

### Identification, heterologous expression and purification of *pipU*, *V*, *W* and *X*

We previously discovered PipA in *R. ruber* No. 14 which cleaves the methylenedioxyphenyl group in piperine. Though piperine is an acceptable substrate for PipA, it is more specific for piperonylic acid, a predicted piperine metabolic intermediate (10). Genome sequencing of *R*. *ruber* No. 14 revealed that *pipM* and *pipA* are located in close proximity. Considering that piperic acid is a product of piperine decomposed by PipM, and that it would be a likely precursor of piperonylic acid, we assumed that other enzymes in *R. ruber* No. 14 catalyze the conversion of piperic acid to piperonylic acid.

**Figure 3:**
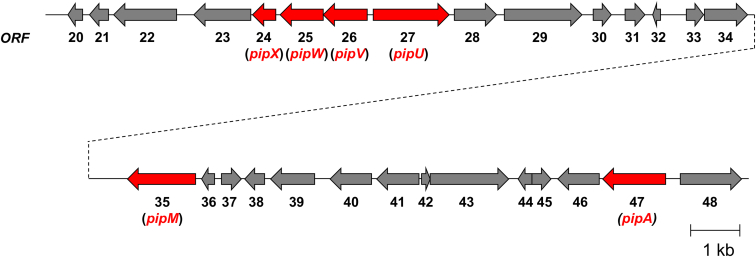
**Partial organization of the piperine-metabolizing gene cluster in *R. ruber* No. 14.** Enzyme-coding genes that we investigated in the previous study (*pipA*) and this study *(pipM, U*, *V*, *W* and *X*) are shown in *red*. Table S2 shows details of the entire cluster.

The mRNA-seq analysis revealed that adding piperine to the culture medium significantly upregulated the transcription of 42 of 48 genes (including *pipM*) that were closely located (Fig. 3 and Table S2). Database analysis and amino acid sequence alignment indicated that in these induced genes, the deduced amino acid sequences derived from four contiguous genes (*pipU***,** *V*, *W* and *X*) (Figs. S12–S15) were similar to enzymes involved in the *p*-hydroxycinnamates (*p*HCAs) metabolism (Figs. S16–S19 and Table S4) (22, 23). *p*HCAs are aromatic compounds with C3 side-chains attached to a benzene ring, instead of the C5 side-chain in piperic acid. In the *p*HCAs metabolism, the side-chains of substrates are metabolized *via* a β-oxidation-like pathway, forming *p*-benzoates and acetyl-CoA. Given that piperic acid and *p*HCAs are similar in structure, we speculated that PipU, V, W and X would catalyze the reaction from piperic acid to piperonylic acid *via* a β-oxidation-like pathway similarly to the *p*HCAs metabolism (Fig. S20*A*). Thus, each of *pipU***,** *V*, *W*, and *X* was cloned and expressed in *Escherichia coli*, and the recombinant enzymes were purified (Fig. S21).

### Characterization of PipU, V, W, and X in piperine metabolism

Incubating piperic acid with PipU, V, W and X generated two products (**5** and **6)** on HPLC (Fig. 1*D*). Reactions that were stopped at 0 min were used as controls for comparison. Analysis by LC-MS in the negative mode revealed that products **5** and **6** respectively peaked at m/z 165 [M-H]^−^ and m/z 191 [M-H]^−^ (Fig. S22). According to our speculation, if piperic acid is degraded similarly to the *p*HCAs metabolism, it should be initially converted to the intermediate, 3,4-methylenedioxycinnamic acid then further degraded into piperonylic acid (Fig. S20*A*). The calculated molecular masses of these compounds were 192 and 166, respectively, which matched those of the detected products. The LC-MS analysis showed that the reaction times and UV spectra of authentic piperonylic acid and 3,4-methylenedioxycinnamic acid respectively corresponded to those of products **5** and **6** (Fig. S23). Thus, we identified product **5** as piperonylic acid and product **6** as 3,4-methylenedioxycinnamic acid. Specialized HPLC that detects acetyl-CoA revealed that it accumulated along with the formation of 3,4-methylenedioxycinnamic acid and piperonylic acid (Fig. S24).

### Functions of PipU, V, W, and X in piperine metabolism

Based on the putative role and the sequence similarity with the enzymes that are involved in the *p*HCAs metabolism, we predicted that PipU would initially conjugate CoA with piperic acid. We therefore incubated piperic acid with CoA, ATP and purified PipU. The LC-MS analysis showed that piperic acid decreased as an unknown product formed at m/z 966 [M-H]^−^ (Fig. S25). This value corresponded to a molecular mass of 967, which was consistent with the predicted mass of CoA-conjugated piperic acid. These findings supported that PipU catalyzes the conjugation of CoA to piperic acid (Fig. S20*A*).

Following the reaction of PipU, we predicted that PipX and PipV would oxidize the β-carbon atom *via* consecutive hydration and dehydrogenation steps. However, LC-MS analysis did not detect additional product peaks when PipX alone or with PipV were included in reaction mixtures that contained PipU (Fig. S26). Although we were not able to detect the products of PipX or PipV, 3,4-methylenedioxycinnamic acid and piperonylic acid did not form if any one of the PipU, V, W and X was removed from the reaction mixtures (Fig. S27). These findings indicated that all of these enzymes are required in the metabolism of piperic acid.

### Substrate specificity of PipU, V, W, and X

**Figure 4:**
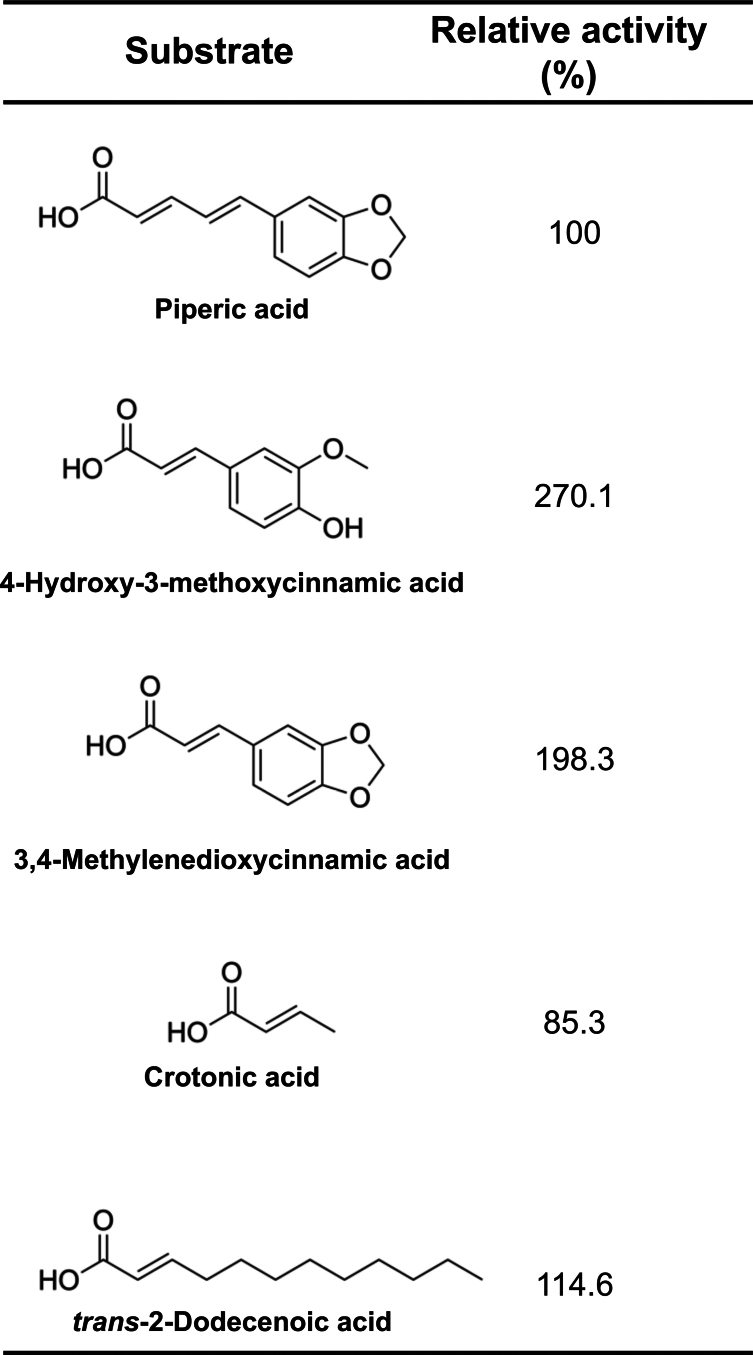
**Substrate specificity of PipU, V, W and X.** Activities of PipU, V, W and X towards candidate substrates were calculated as acetyl-CoA accumulation. PipU, V, W and X can catalyze both fatty acids and aromatic compounds but prefers *p*HCAs.

We investigated the substrate specificity of the PipU, V, W and X using the following potential substrates. We assessed how the side-chain lengths of the aromatic compounds affect the activities of PipU, V, W and X using piperic acid (C5 side-chain) and two *p*HCAs (4-hydroxy-3-methoxycinnamic acid and 3,4-methylenedioxycinnamic acid; C3 side-chain). We also investigated whether fatty acids could be potential substrates to PipU, V, W and X or not. Two fatty acids, which have an unsaturated C=C bond on the β-carbon atom that is similar to the side-chain of *p*HCAs (crotonic acid and *trans*-2-dodecenoicacid acid) were selected (Fig. 4). We assessed substrate availability based on the accumulation of acetyl-CoA, which is a key metabolic product in β-oxidation. LC-MS analysis revealed the acetyl-CoA accumulation in each of the reaction mixtures (Fig. S28), indicating that PipU, V, W and X act on all tested substrates. Using piperic acid as the reference (100%), the relative activities of PipU, V, W and X on 4-hydroxy-3-methoxycinnamic acid, 3,4-methylenedioxycinnamic acid, crotonic acid and *trans*-2-dodecenoic acid were 270.1%, 198.3%, 85.3% and 114.6%, respectively (Fig. 4). These findings demonstrated that PipU, V, W and X can decompose both fatty acids and aromatic compounds but prefer *p*HCAs.

## Discussion

Pepper is one of the most important seasonings due to its characteristic spiciness and health-promoting bioactive properties, accounting for ∼ 34% of global spice trade, with an annual production of around 450,000 tons (24). Piperine, the major alkaloid produced by black pepper, has a distinctive structure that differs from most other plant-derived secondary metabolites. Notably, while tertiary amide bonds in alkaloids are typically embedded within heterocyclic carbon-nitrogen rings (Fig. 1*B*), of which microbial metabolism has been reported (5), piperine’s tertiary amide bond is formed between heterocyclic amine and fatty acid side chain (Fig. 1*A*), which is a rare structure among natural alkaloids. Meanwhile, though direct research about how piperine interferes the constitution of local microbial communities is lacking, it might play essential roles in the interspecies interaction of microorganisms and pepper trees when considering its multiple antibiotic effects (4). Therefore, elucidating microbial piperine metabolism is of particular interest, as it may reveal novel enzymatic mechanisms and provide insights into interactions between microorganisms and pepper trees.

To unveil microbial piperine metabolism, we started to screen piperine-metabolizing microorganisms. Soil microorganisms that have specific metabolizing abilities could be found in such conditions (9, 10). Previously, we isolated the piperine-degrading actinomycete, *R. ruber* No. 14, from soil samples collected from areas of Okinawa where *P. nigrum* grows (10).

**Figure 5:**
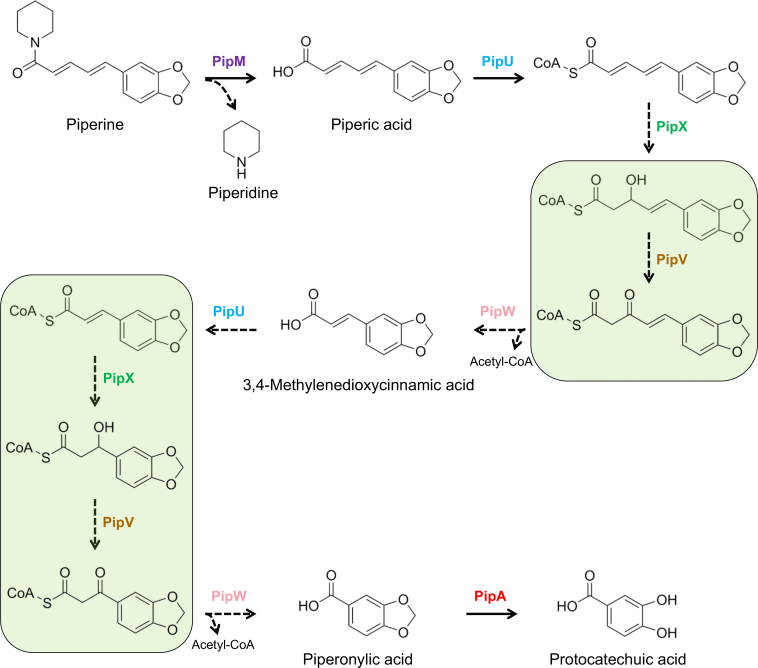
**Proposed piperine-metabolizing pathway in *R. ruber* No. 14.***Solid arrows* indicate the confirmed reaction steps, and *dashed arrows* indicate the proposed reaction steps. The *green squares* indicate the predicted intermediates that have not been detected by LC-MS.

**Figure 6:**
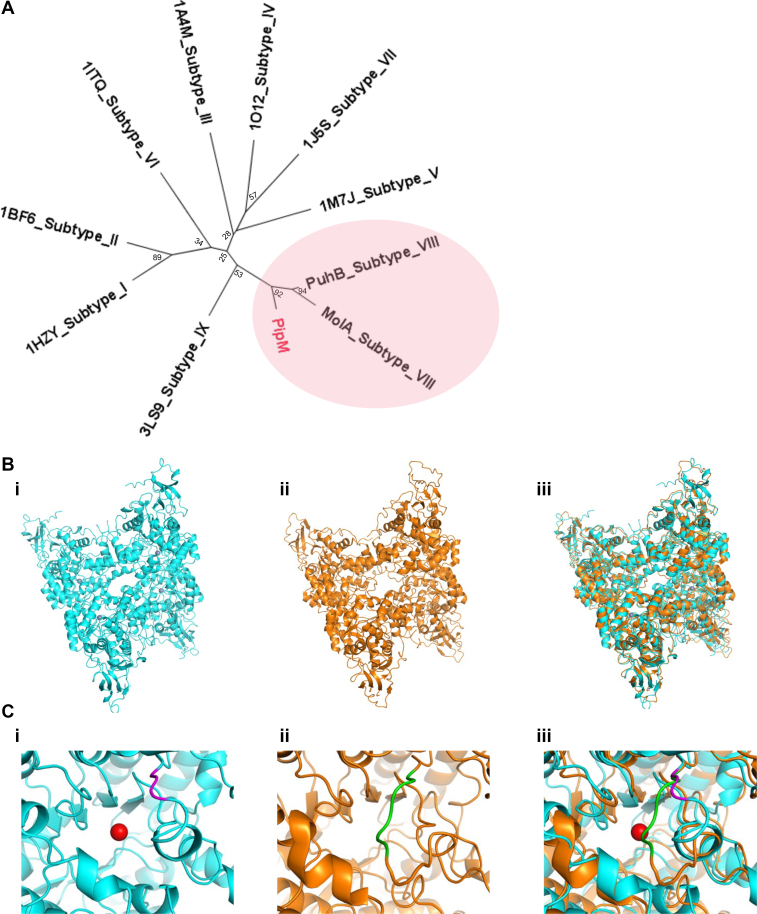
**Comparison of PipM and AHS enzymes.***A*, phylogenetic analysis of PipM regarding the selected AHS enzymes. Amino acid sequences of AHS enzymes that are representative of each subtype: subtype I, PDB code 1HZY; subtype II, PDB code 1BF6; subtype III, PDB code 1A4M; subtype IV, PDB code 1O12; subtype V, PDB code 1M7J; subtype VI, PDB code 1M7J; subtype VII, PDB code 1J5S; subtype VIII, PuhB (PDB code 4WHB), MolA (PDB code 4WGX) and PipM; subtype IX, PDB code 3LS9. PipM and two other subtype VIII enzymes form a single clade (*shaded pink*). *B*, structure comparison of PipM to PuhB. (i) Simulated structure of PipM; (ii) structure of PuhB (PDB code 4WHB); (iii) molecular overlap of PipM to PuhB. *C*, lid-like Structure in PipM and PuhB. (i) Lid-like structure of PipM; (ii) lid-like structure of PuhB; (iii) overlap of lid-like structure in PipM and PuhB. *Red*, predicted Zn^2+^ ion; *purple and green*, lid-like structures in PipM and PuhB, respectively. Compared with PuhB, the simulated PipM lacks a lid-like structure, resulting in a larger entrance of its active site.

From this strain, here, we isolated piperine hydrolase (PipM), which specifically catalyzes the hitherto unknown hydrolysis of acyclic tertiary amide structure (Fig. 5). Due to the absence of a signal peptide, PipM likely acts on piperine after it has been taken up by strain No. 14 from the surrounding environment. The relatively low *K*_*m*_ value of PipM (2.11 ± 0.31 μM) suggests that strain No. 14 is physiologically capable of utilizing piperine. Based on BLAST search, we identified PipM belongs to the amidohydrolase superfamily (AHS), a group of enzymes that catalyze the hydrolysis of various bonds, including C-O, P-O, C-N, C-S, and even C-Cl (20, 21, 25, 26). All AHS enzymes are reported to have a metal-binding site located next to their active sites (27). Recent studies classified the AHS enzymes into nine subtypes based on conserved residues that surround the metal-binding sites (20, 27, 28). A BLAST search revealed three PipM homologues, PuhA, PuhB, and MolA, all of which have been classified as subtype VIII AHS enzymes (29, 30). Among these homologues, PuhB and MolA, respectively, hydrolyze the C-N bond in diuron and the C-S bond in molinate (Fig. S29) (20, 21). The crystal structures of these enzymes have been determined (30). Among all AHS enzymes, subtype VIII shares a characteristic conversed metal-binding motif that is consisted by Asn-X-His-X-Lys-X-His-X-His-X-Asp (30). Multiple-sequence alignment of PipM with subtype I to IX AHS enzymes revealed that PipM has the same constitution of residues in the metal-binding motif as other subtype VIII AHS enzymes (Fig. S30). Meanwhile, the findings of our metal analysis of PipM were consistent with the fact that Subtype VIII AHS enzymes are Zn^2+^ or Co^2+^ dependent. Moreover, PipM was found to be a homotetramer, which was consistent with PuhB and MolA (30). Further phylogenetic analysis also showed that PipM shares a higher homology with PuhB and MolA than with enzymes from other subtypes (Fig. 6*A*). These findings indicated that PipM is a new member of subtype VIII AHS.

To further compare PipM with other subtype VIII AHS enzymes, we used AlphaFold3 to predict the PipM structure and compared it with PuhB, its closest homologue. The predicted structure of PipM had close identity with that of PuhB in three dimensions (Fig. 6*B*). Like PuhB, the PipM monomer consists of a (β/α)_8_ barrel domain and an accessory β sandwich domain in the *N*-terminal. The (β/α)_8_ barrel domain of four monomers were oriented inward, forming a twisted cross-like configuration (Fig. 6*B*). However, structures differed between the two enzymes in the unregulated loop regions. The loop structures in AHS enzymes might play crucial roles in substrate specificity (27, 31). A part of the loop in PuhB forms a lid-like structure near the metal-binding site, which partially blocks the entrance to the active site, thus preventing access by large molecules (Fig. 6*C*). In the counterpart of PipM, three amino acids were depleted, resulting in a smaller loop near the active-site entrance (Fig. 6*C* and Fig. S31). This structural variation might explain why PipM can accommodate larger substrates such as piperine and piperlongumine, both of which have a tertiary amide bond in the structure. Considering that PipM was completely inert to small molecules such as diuron and molinate, this enzyme might not stably bind small molecules but specialize in larger compounds. These findings demonstrated that PipM plays an important role in *R*. *ruber* No. 14, which can catabolize piperine as its sole nitrogen source, for metabolism of piperine and piperlongumine derived from pepper fruit in nature.

We analyzed the entire genome of *R*. *ruber* No. 14 and performed mRNA-seq using bacteria cells obtained from culture media with or without piperine. We found that the transcription of 42 of 48 genes that were physically located close to each other on the genome, including *pipM*, increased ∼ 20-fold in the presence of piperine. Notably, PipA, a methylenetransferase we previously found that acts on the methylenedioxyphenyl group of piperine and piperine derivatives, was also one of these piperine-induced genes. These findings indicated the existence of a piperine-metabolizing related gene cluster (Fig. 3 and Table S2). Given that *R*. *ruber* No. 14 was isolated from medium in which piperine was the sole source of nitrogen, these induced genes must be important for this strain to catabolize piperine. We previously speculated that piperine would be somehow converted into piperonylic acid before PipA could function (10). Since the present findings confirmed that PipM catabolizes piperine into piperic acid and piperidine, we started to search other genes encoding enzymes that might be involved in the subsequent reaction from piperic acid to piperonylic acid in this gene cluster.

Database analyses revealed that in the piperine-metabolizing gene cluster, the deduced amino acid sequences of four contiguous genes, *pipU*, *V*, *W* and *X*, were similar to those of enzymes involved in the metabolism of *p*HCAs in *Rhodococcus jostii* RHA1 and *Agrobacterium fabrum* sp. (22, 26). The *p*HCAs metabolism follows a β-oxidation-like pathway but differs significantly from the conventional β-oxidation in key aspects. In conventional β-oxidation, the reaction begins with the conjugation between fatty acids and CoA, followed by sequential dehydrogenation, hydration, oxidation and thiolysis steps that are catalyzed by a series of enzymes, resulting in the production of acyl-CoA and acetyl-CoA (Fig. S20*C*) (32). The enzymes responsible for the hydration, oxidation, and thiolysis steps usually form a tri-functional enzyme (TFE) complex (33). In contrast, an aromatic carboxylic acid is produced in the *p*HCAs metabolism instead of acyl-CoA in the thiolysis step during conventional β-oxidation (Fig. S20, *B* and *C*) (22, 23). Furthermore, deletion of the gene encoding the enzyme responsible for the oxidation step does not affect the hydration step during the *p*HCAs metabolism (22), suggesting that enzymes do not form a complex.

We found that PipU, V, W and X catalyzed the conversion of piperic acid into piperonylic acid *via* a *p*HCAs intermediate 3,4-methylenedioxycinnamic acid (Fig. S20*A*), suggesting that piperic acid is metabolized through a β-oxidation-like pathway, similar to the *p*HCAs metabolism in *R*. *jostii* RHA1 and *A. fabrum* sp. Because the side-chain is longer in piperic acid than in *p*HCAs (Fig. S20), we next investigated the substrate specificity of PipU, V, W and X. The activity of the four enzymes was ∼2-fold that towards 4-hydroxy-3-methoxycinnamic acid and 3,4-methylenedioxycinnamic acid than piperic acid, indicating a clear preference for *p*HCAs. Furthermore, we found that PipU, V, W, and X could degrade fatty acids (Fig. 4), indicating that these enzymes have a broader substrate range than originally expected. Such metabolic versatility could confer a significant survival advantage to bacterial strains expressing these enzymes, particularly in isolated environments such as Ishigaki Island, Okinawa.

BLAST analysis revealed that PipU shares ∼ 50% sequence similarity with the acyl-CoA synthetase associated with β-oxidation in *Pseudomonas* and *Mycobacterium* strains (Table S5) (34, 35). However, PipV, W and X was not significantly similar to the TFE complex identified from strains in these genera (Tables S6–S8) (36, 37, 38). These findings suggested that while *p*HCAs metabolism and fatty acid β-oxidation start similarly, the enzymes responsible for the chain cleavage might function in a completely different mechanism (Fig. S20).

In conclusion, we discovered piperine hydrolase, which decomposes piperine into piperic acid and piperidine, as well as other piperine-metabolizing enzymes that catalyze the subsequent conversion of piperic acid to piperonylic acid. Along with our previous study, we have clarified the metabolic pathway from piperine to protocatechuic acid (Fig. 5). Since protocatechuic acid can be fully degraded into acetyl-CoA in some bacteria (39), *R. ruber* No. 14 may metabolize piperine primarily as an energy source. Given piperine’s defensive function in pepper plants, the presence of *R. ruber* No. 14 may pose a threat to the growth of this important crop. Additionally, our study sheds light on understanding how a soil microorganism interacts with this important commercial crop in semi-tropical or tropical areas. Certain intermediates generated during piperine metabolism may participate in various ecological interactions—between the black pepper plant and piperine-degrading microorganisms, between these microorganisms and other symbiotic microbes in the rhizosphere, and between the degraders and plant-antagonistic microbial species. Functional assays on these intermediates after their overproduction and isolation could provide further insights into these complex interactions.

Our present study unveiled the natural microbial piperine metabolism, providing insights into the hitherto unknown microbial metabolism of the alkaloids with acyclic tertiary amide structures. Given the wide range of substrate specificity of PipU, V, W and X, these enzymes may convert various aromatic substrates that have a long side-chain into analogues of *p*HCAs and piperonylic acid, which are possible precursors of many high value chemicals with pharmacological potentials (*e.g.* sinapic acid and sesamol; (40, 41)), our results also contributed to the theoretical foundation of *in vitro* production of these value-added chemicals. Moreover, this study also emphasized that actinomycetes play important roles in ecosystems by not only synthesizing diverse secondary metabolites but also degrading complex natural organic compounds. Therefore, clarifying how actinomycetes utilize plant-derived specialized metabolites could provide new insights into the universal understanding of their roles in the nutrient cycle and the ecological balance.

## Experimental procedures

### Chemicals

Chemicals were purchased from the following suppliers: RbCl, CsCl, Na_2_MoO_4_·2H_2_O, CoCl_2_·6H_2_O, 5,5′-dithio-bis-2-nitrobenzoate (DTNB), iodoacetate, hydroxylamine·HCl, phenylhydrazine·HCl, semicarbazide·HCl, *o*-phenanthroline·H_2_O, KCN, diisopropyl fluorophosphate (DFP), nicotinamide adenine dinucleotide (NAD), CoA, molinate, crotonic acid and piperine (Wako Pure Chemical Industries, Ltd, Osaka, Japan); piperic acid, piperonylic acid, 3,4-methylenedioxycinnamic acid, piperlongumine, 3-(3,4-dichlorophenyl)-1,1-dimethylurea (diuron), *N*,*N*-dimethylbutylamine (DMBA), 4-fluoro-7-nitro-2,1,3-benzoxadiazole (NBD-F), aminoguanidine·H_2_CO_3_ and *trans*-2-dodecenoic acid (Tokyo Chemical Industries); formic acid, methanol, LiCl, PbCl_2_, NaCl, HgCl_2_, MgCl_2_·6H_2_O, NiCl_2_·6H_2_O, CaCl_2_·2H_2_O, CuCl_2_·2H_2_O, BaCl_2_·2H_2_O, FeSO_4_·7H_2_O, MnCl_2_·4H_2_O, FeCl_3_, ZnCl_2_, CdCl_2_·2.5H_2_O, SrCl_2_·6H_2_O, AlCl_3_·6H_2_O, *N*-ethylmaleimide (NEM), *p*-chloromercuribenzoate (PCMB), *α*,*α*′-dipyridyl, 8-hydroxyquinoline, ethylenediaminetetraacetic acid (EDTA), NaN_3_, dithiothreitol (DTT), diethyldithiocarbamate·3H_2_O, 2-mercaptoethanol, sodium hydrosulfate (Na_2_S_2_O_4_), H_2_O_2_, ammonium persulfate, phenylmethanesulfonyl fluoride (PMS), adenosine triphosphate (ATP), acetyl-CoA and 4-hydroxy-3-methoxycinnamic acid (Nacalai Tesque Co., Inc.).

### Bacterial strains, plasmids, and primers

*R. ruber* No. 14 was isolated from detritus soil sample collected in Ishigaki Island, Okinawa, Japan (10). Other bacterial strains, plasmids, and primers are shown in Table S1.

### Preparation of *R. ruber* no. 14 culture supernatants

For the investigation of enzyme activity in the culture supernatants, *R. ruber* No. 14 was first inoculated into 5 ml of 2 × Yeast Extract Tryptone (YT) medium and incubated at 28 °C for 72 h. Subsequently, this 5 ml preculture broth was transferred into 100 ml of fresh 2 × YT medium supplemented with 0.01% (wt/vol) piperine to incubate at 28 °C for 72 h. The resultant culture broth was centrifuged at 5840*g* for 15 min, then the supernatants were decanted and passed through a MILLEX HV 0.45 μm filter to remove bacterial cells.

For purification of the culture supernatants, *R. ruber* No. 14 was first inoculated into 5 ml of 2 × YT medium and incubated at 28 °C for 72 h. Subsequently, 2 ml of this preculture broth was transferred into 200 ml of fresh 2 × YT medium supplemented with 0.01% (wt/vol) piperine to incubate at 28 °C for 72 h. Thereafter, 12 portions (10 ml each) of the resultant culture broth were then individually inoculated into 1 L of medium containing piperine as the sole nitrogen source to incubate at 28 °C for 72 h. Finally, a total of 12 L of this culture broth was centrifuged at 10,000*g* for 20 min, and the resultant supernatants were recovered for further analysis.

### Purification of PipM from R. ruber No. 14 culture supernatants

We purified PipM from culture supernatants as follows.

Step 1: We stirred supernatants of the 12 L culture broth with 150 ml of DEAE Sepharose (GE Healthcare) which is equilibrated with 20 mM Tris-HCl (pH 8.0) overnight then packed them into a column. After washing the resultant column with 200 ml of 20 mM Tris-HCl (pH 8.0), proteins were sequentially eluted using 200 ml each of the following buffers: 100 mM NaCl/20 mM Tris-HCl (pH 8.0), 250 mM NaCl/20 mM Tris-HCl (pH 8.0), 500 mM NaCl/20 mM Tris-HCl (pH 8.0), and 1 M NaCl/20 mM Tris-HCl (pH 8.0). We recovered 800 ml of enzyme samples and added (NH_4_)_2_SO_4_ into the samples to reach 80% saturation, then stirred them on ice for 1 h and centrifuged at 15,700*g* for 10 min. Precipitated proteins were recovered in 40 ml of 20 mM Tris-HCl (pH 8.0).

Step 2: 40 ml of the recovered protein samples were passed through a HiPrep Butyl 20 ml column (GE Healthcare). Buffers A and B, respectively, comprised 50 mM K_2_HPO_4_/KH_2_PO_4_ (pH 7.5) and 1 M (NH_4_)_2_SO_4_/50 mM K_2_HPO_4_/KH_2_PO_4_ (pH 7.5). The column was equilibrated with 60 ml of 50 mM K_2_HPO_4_/KH_2_PO_4_ (pH 7.5), and then the enzymes were eluted with a 0% to 100% gradient of buffer B and dialyzed to remove (NH_4_)_2_SO_4_.

Step 3: Fractions of HiPrep Butyl 20 ml column that with piperine-metabolizing activity were applied to a Resource Q 6 ml column (GE Healthcare). Buffers A and B, respectively, comprised 50 mM K_2_HPO_4_/KH_2_PO_4_ (pH 7.5) and 1 M NaCl/50 mM K_2_HPO_4_/KH_2_PO_4_ (pH 7.5). The column was equilibrated with 18 ml of 50 mM K_2_HPO_4_/KH_2_PO_4_ (pH 7.5), and then enzymes were eluted with an increasing gradient of buffer B (0%–100%).

Step 4: Fractions of Resource Q 6 ml column that with piperine-metabolizing activity were applied to a BioAssist Q 1 ml column (Tosoh Bioscience, Tokyo, Japan). Buffers A and B, respectively, comprised 50 mM K_2_HPO_4_/KH_2_PO_4_ (pH 7.5) and 1 M NaCl/50 mM K_2_HPO_4_/KH_2_PO_4_ (pH 7.5). The column was equilibrated with 3 ml of 50 mM K_2_HPO_4_/KH_2_PO_4_ (pH 7.5), and then enzymes were eluted with a 0% to 100% gradient of buffer B (%).

Steps 2 to 4 proceeded using an AKTA purifier (GE Healthcare).

### Determination of protein concentration

Protein concentrations were determined spectrophotometrically at 280 nm using a BioSpec-nano spectrophotometer (Shimadzu Corp). The molar extinction coefficients of each enzyme were calculated by the following WEB site (https://web.expasy.org/protparam/).

### Prediction of signal peptide in PipU

The signal peptide prediction for PipM was performed using SignalP 6.0 (42), https://services.healthtech.dtu.dk/services/SignalP-6.0/).

### mRNA-sequence

We inoculated *R. ruber* No. 14 in 5 ml of 2 × YT media supplemented without or with 0.01% (w/v) piperine and incubated at 28 °C for 48 h. Total mRNA extracted using the NucleoSpin RNA kit (Takara Bio Inc) was analyzed using an Illumina NGS (Illumina Inc.).

### Assays of PipM activity

For the investigation of piperine degradation activity in *R. ruber* No. 14 culture supernatants, the assays were performed in 100 μl total volume containing 0.2 mM piperine, 50 mM K_2_HPO_4_/KH_2_PO_4_ (pH 7.5), and 0.00164 mg/ml of the culture supernatants. The reactions were carried out at 28 °C for 3 h.

For the investigation of PipM activities at all steps of culture supernatants purification, the assays were performed in 100 μl total volume containing 0.2 mM piperine, 50 mM K_2_HPO_4_/KH_2_PO_4_ (pH 7.5), and PipM (0.0112 μM, 0.00766 μM, 0.0167 μM, and 0.0208 μM). The reactions were carried out at 28 °C for 10 min.

For the investigation of the activities of recombinant PipM, the assays were performed in 100 μl total volume containing 0.2 mM piperine, 50 mM K_2_HPO_4_/KH_2_PO_4_ (pH 7.5), and 0.116 μM PipM. The reactions were carried out at 28 °C for 10 min.

### Gene cloning and heterologous enzyme expression

We amplified the *pipM, U*, *V*, *W* and *X* genes by PCR under the following conditions: 34 cycles of 94 °C for 120 s, 98 °C for 10 s, 68 °C for 40 s, using the primers shown in Table S2. The PCR products were cloned into the linearized pET24a(+) vector using In-Fusion HD Cloning Kits (Clontech Laboratories Inc.). The resulting plasmids were referred to as pET24a(+)-*pipM*, pET24a(+)-*pipXhis*, pET24a(+)-*pipW*, pET24a(+)-*pipVhis* and pET24a(+)-*pipUhis*. *E. coli* Rosetta2 (DE3) cells were individually transformed with each plasmid and cultured in 1 L of 2 × YT medium (containing 50 μg/ml each of kanamycin and chloramphenicol) at 37 °C for 2 h. The cells were incubated with a final concentration of 0.5 mM in isopropyl-β-D-thiogalactoside (IPTG) for 16 h at 18 °C. Cells were separated from the broth by centrifugation at 5640*g* at 4 °C for 15 min, resuspended in 30 ml of 50 mM K_2_HPO_4_/KH_2_PO_4_ (pH 7.5) and disrupted using an Ultrasonic Generator (Kubota Corporation Co., Ltd). The lysate was centrifuged for 15 min at 4 °C and 16,200*g*, and then the recovered supernatants (cell-free extract) were purified.

### Purification of recombinant enzymes

Recombinant enzymes were purified using the ӒKTA pure column chromatography system (GE Healthcare) as follows.

#### PipM

Step 1: Cell-free extracts were applied to a HiPrep DEAE FF 20 ml column (GE Healthcare). Buffer A and B were 50 mM K_2_HPO_4_/KH_2_PO_4_ (pH 7.5) and 1 M NaCl/50 mM K_2_HPO_4_/KH_2_PO_4_ (pH 7.5), respectively. The column was equilibrated with 60 ml of 50 mM K_2_HPO_4_/KH_2_PO_4_ (pH 7.5), and then enzyme was eluted with a 0% to 100% gradient of buffer B.

Step 2: Fractions of step 1 that showed piperine-metabolizing activity were applied to a Resource Q 6 ml column (GE Healthcare). Buffer A and B were 50 mM K_2_HPO_4_/KH_2_PO_4_ (pH 7.5) and B 1 M NaCl/50 mM K_2_HPO_4_/KH_2_PO_4_ (pH 7.5), respectively. The column was equilibrated with 18 ml of 50 mM K_2_HPO_4_/KH_2_PO_4_ (pH 7.5), and then enzyme was eluted with a 0% to 100% gradient of buffer B.

Step 3: Fractions of step 2 that showed piperine-metabolizing activity were applied to a Butyl 650M 5 ml column (Tosoh Bioscience). Buffer A and B were 50 mM K_2_HPO_4_/KH_2_PO_4_ (pH 7.5) and 1 M (NH_4_)_2_SO_4_/50 mM K_2_HPO_4_/KH_2_PO_4_ (pH 7.5), respectively. The column was equilibrated with 15 ml of 50 mM K_2_HPO_4_/KH_2_PO_4_ (pH 7.5), and then enzyme was eluted with a 0%–100% gradient of buffer B. Fractions with piperine-metabolizing activity were retrieved and dialyzed to remove (NH_4_)_2_SO_4_.

#### PipX, PipV, and PipU

Cell-free extracts were applied to Histrap HP 5 ml columns (GE Healthcare). Buffers A and B were 50 mM K_2_HPO_4_/KH_2_PO_4_ (pH 7.5) and 250 mM imidazole/50 mM K_2_HPO_4_/KH_2_PO_4_ (pH 7.5), respectively. The columns were equilibrated with 15 ml of 50 mM K_2_HPO_4_/KH_2_PO_4_ (pH 7.5), and then enzymes were eluted with an increasing percentage of buffer B (0%**–**100%). Fractions with piperine-metabolizing activity were retrieved.

#### PipW

Cell-free extract was applied to a HiPrep DEAE FF 20 ml column (GE Healthcare). Buffers A and B were 50 mM K_2_HPO_4_/KH_2_PO_4_ (pH 7.5) and 1 M NaCl/50 mM K_2_HPO_4_/KH_2_PO_4_ (pH 7.5), respectively. The column was equilibrated with 60 ml of 50 mM K_2_HPO_4_/KH_2_PO_4_ (pH 7.5), and then enzyme was eluted with an increasing percentage of buffer B (0%**–**100%). Fractions with piperine-metabolizing activity were retrieved.

### Identification of piperidine

To confirm that piperidine is a reaction product of PipM, the assays were performed in 100 μl total volume containing 0.15 mM piperine, 15.7 mM EDTA, and 0.00328 mg/ml of the culture supernatants. The reactions were carried out at 28 °C for 18 h. Thereafter, 1 μl of 100 mM NBD-F (in acetonitrile) and 99 μl of 50 mM HCl were added to the reaction mixture, and LC-MS analysis was used for product identification.

### Molecular mass determination of native PipM

We determined the molecular mass of PipM using gel-filtration chromatography. Concentrated PipM samples (300 μl; 4.4 mg/ml) were eluted through a Superdex200 Increase 10/300 Gl column (GE Healthcare) with 0.1 M NaCl/50 mM K_2_HPO_4_/KH_2_PO_4_ (pH 7.5) using the AKTA purifier (GE Healthcare) at a flow rate of 2 mL min^−1^. Standard glutamate dehydrogenase (290 kDa), lactate dehydrogenase (142 kDa), enolase (67 kDa), myokinase (32 kDa), and cytochrome *c* (12.4 kDa) proteins (MW-Marker [HPLC], Oriental Yeast Co., Ltd) were eluted before and after sample injection. The molecular mass of PipM was calculated based on the mobility of the standard proteins.

### Temperature dependency and stability of PipM

We estimated thermal dependency by incubating reaction mixtures (100 μl) containing 0.116 μM PipM, 0.01 mM piperine and 50 mM K_2_HPO_4_/KH_2_PO_4_ (pH 7.5) at a temperature range of 10 °C–80 °C for 10 min. Thermal stability was estimated by incubating PipM for 20 min at 10 °C–80 °C in a Dry Thermo Unit DTU-1B (Taitec Corp.) then adding it to the reaction mixture as described for thermal dependency, followed by incubation for 10 min at 28 °C. The amounts of reaction products were determined by high-performance liquid chromatography with photodiode array detection (HPLC-PDA). All assessments proceeded in triplicate.

### pH dependency and stability PipM

pH dependency and stability were estimated as follows. For pH dependency, reaction mixtures containing 0.116 μM PipM and 0.01 mM piperine were adjusted to pH 2.0 to 12.0 using 0.4 M Britton-Robinson buffer (pH 2.0–12.0 [1.0 pH units]) and then adjusted to 100 μl using diluted water. Thereafter, the reaction mixtures were incubated at 28 °C for 10 min.

For pH stability, PipM (1.18 mg/ml; 2.32 μM) was pre-incubated on ice for 30 min in 0.4 M Britton-Robinson buffer (pH 2.0–12.0 [1.0 pH units]). Using 5 μl of these pre-incubated PipM, we then prepared 100 μl of reaction mixtures containing 0.116 μM of pre-incubated PipM, 0.01 mM piperine and 50 mM K_2_HPO_4_/KH_2_PO_4_ (pH 7.5); they were incubated at 28 °C for 10 min. Reaction products were quantified using HPLC-PDA. All assessments proceeded in triplicate.

### Kinetic assay of PipM

Reaction mixtures (100 μl) comprising 0.116 μM PipM,0.001 to 0.5 mM piperine and 50 mM K_2_HPO_4_/KH_2_PO_4_ (pH 7.5) were incubated at 28 °C for 1 min. The velocity of piperic acid formation was calculated based on HPLC analysis at 340 nm. One unit of enzyme activity was defined as the amount of enzyme required to catalyze the formation of 1 μmol/min of piperic acid.

### Metal analysis of PipM

Concentrated PipM samples (8 ml, 4.18 mg/ml) were dialyzed in 50 mM K_2_HPO_4_/KH_2_PO_4_ (pH 7.5). The inner dialysates were adjusted to a final concentration of 40.1 μM in 12 ml of 50 mM K_2_HPO_4_/KH_2_PO_4_ (pH 7.5). Metal-free PipM and its outer dialysates were analyzed using an inductively coupled plasma (ICP) optical emission spectrometer (ICPS-8100; Shimadzu).

### Effect of inhibitors on PipM activity

We assessed the effects of the metal inhibitors LiCl, PbCl_2_, NaCl, HgCl_2_, MgCl_2_·6H_2_O, NiCl_2_·6H_2_O, CaCl_2_·2H_2_O, CuCl_2_·2H_2_O, BaCl_2_·2H_2_O, FeSO_4_·7H_2_O, MnCl_2_·4H_2_O, FeCl_3_, ZnCl_2_, RbCl, CdCl_2_·2.5H_2_O, SrCl_2_·6H_2_O, CoCl_2_·6H_2_O, CsCl, AlCl_3_·6H_2_O, Na_2_MoO_4_·2H_2_O and the non-metal inhibitors 5,5′-dithio-bis-2-nitrobenzoate (DTNB), iodoacetate, *N*-ethylmaleimide (NEM), *p*-chloromercuribenzoate (PCMB), hydroxylamine·HCl, phenylhydrazine·HCl, semicarbazide·HCl, aminoguanidine·H_2_CO_3_, *α*,*α*′-dipyridyl, *o*-phenanthroline·H_2_O, 8-hydroxyquinoline, ethylenediaminetetraacetic acid (EDTA), diethyldithiocarbamate·3H_2_O, NaN_3_, KCN, dithiothreitol (DTT), 2-mercaptoethanol, sodium hydrosulfate (Na_2_S_2_O_4_), H_2_O_2_, ammonium persulfate, phenylmethanesulfonyl fluoride (PMS), and diisopropyl fluorophosphate (DFP). Reaction mixtures containing 0.116 μM PipM, 0.2 mM piperine, 1 mM inhibitors were adjusted to 100 μl with 50 mM K_2_HPO_4_/KH_2_PO_4_ (pH 7.5) and incubated at 28 °C for 10 min. Reaction products were quantified in triplicate using HPLC-PDA. All assessments proceeded in triplicate.

### Substrate specificity assay of PipM

Reaction mixtures (100 μl) containing 0.116 μM PipM, 1 mM substrate (piperine, diuron, piperlongumine or molinate) and 50 mM K_2_HPO_4_/KH_2_PO_4_ (pH 7.5) were incubated at 28 °C for 2 h. Decreased substrates and increased product formation were analyzed using LC-MS.

### Assay of PipU, V, W and X effects on piperic acid

#### Overall function of PipU, V, W and X on piperic acid

Reaction mixtures (100 μl) containing 0.562 μM PipU, 0.344 μM PipV, 0.137 μM PipW, 1.90 μM PipX, 1 mM each of CoA, NAD^+^, ATP, piperic acid, and50 mM K_2_HPO_4_/KH_2_PO_4_ (pH 7.5) were incubated at 28 °C for 4 h.

#### Each enzyme

##### PipU

Reaction mixtures (100 μl) containing 1.12 μM PipU, 1 mM each of CoA, NAD^+^, ATP, and piperic acid as well as 50 mM K_2_HPO_4_/KH_2_PO_4_ (pH 7.5) were incubated at 28 °C for 2 h.

##### PipX

Reaction mixtures (100 μl) containing 1.12 μM PipU, 1.90 μM PipX, 1 mM each of CoA, NAD^+^, ATP, and piperic acid as well as50 mM K_2_HPO_4_/KH_2_PO_4_ (pH 7.5) were incubated at 28 °C for 4 h.

##### PipV

Reaction mixtures (100 μl) containing 1.12 μM PipU, 0.344 μM PipV, 1.90 μM PipX, 1 mM) each of CoA, NAD^+^, ATP, and piperic acid as well as 50 mM K_2_HPO_4_/KH_2_PO_4_ (pH 7.5) were incubated at 28 °C for 4 h.

#### Requirement for PipU, V, W and X

##### PipX

Reaction mixtures (100 μl) consisting of 0.562 μM PipU, 0.562 μM PipV, 0.137 μM PipW, 1 mM each of CoA, NAD^+^, ATP, and piperic acid as well as 50 mM K_2_HPO_4_/KH_2_PO_4_ (pH 7.5) were incubated at 28 °C for 4 h.

##### PipW

Reaction mixtures (100 μl) consisting of 0.562 μM PipU, 0.344 μM PipV, 1.90 μM PipX, 1 mM each of CoA, NAD^+^, ATP, and piperic acid as well as 50 mM K_2_HPO_4_/KH_2_PO_4_ (pH 7.5) were incubated at 28 °C for 4 h.

##### PipV

Reaction mixtures (100 μl) consisting of 0.562 μM PipU, 0.137 μM PipW, 1.90 μM PipX, 1 mM each of CoA, NAD^+^, ATP, and piperic acid as well as 50 mM K_2_HPO_4_/KH_2_PO_4_ (pH 7.5) were incubated at 28 °C for 4 h.

#### Substrate specificity of PipU, V, W and X

Reaction mixtures (100 μl) comprising 0.562 μM PipU, 0.344 μM PipV, 0.137 μM PipW, and 1.90 μM PipX, 1 mM each of CoA, NAD^+^, ATP, 1 mM substrate (piperic acid, 4-hydroxy-3-methoxycinnamic acid, 3,4-methylenedioxycinnamic acid, crotonic acid or *trans*-2-dodecenoic acid) and 50 mM K_2_HPO_4_/KH_2_PO_4_ (pH 7.5) were incubated at 28 °C for 18 h.

### HPLC analysis

We investigated PipU, V, W and X substrate specificity as the amount of acetyl-CoA detected using 4.6 × 150 mm Cosmosil 5C_18_-AR-II columns (Nacalai Tesque Co., Inc.) and the Prominence HPLC system with an SPD-M10 A Diode array detector (both from Shimadzu). The mobile phases specialized for acetyl-CoA detection were (A) 100 mM NaH_2_PO_4_/75 mM CH_3_COONa and (B) 100% acetonitrile (43). The LC flow rate was 1 mL min^−1^ and the column temperature was 40 °C. The columns were equilibrated with 6% phase B for 5 min, followed by isocratic system (phase A:B = 94:6) for 15 min, linear gradient of 6%–80% (phase B) for 5 min, and 80% methanol for 5 min.

### LC-MS analysis

All LC-MS analyses were proceeded using 4.6 × 150 mm Cosmosil 5C_18_-AR-II columns (Nacalai Tesque Co., Inc. Kyoto, Japan) and the prominence system consisted of a photodiode array detector (SPD-M20 A) and an LCMS-8030 instrument (Shimadzu).

LC/MS/MS analysis was proceeded using 4.6 × 150 mm Cosmosil 5C_18_-AR-II columns (Nacalai Tesque Co., Inc. Kyoto, Japan) and the prominence system consisted of a photodiode array detector (SPD-M20 A) and an LCMS-8030 instrument (Shimadzu) equipped with electrospray ionization (ESI) source. Samples were analyzed in positive ion mode using the following conditions: nebulizer gas flow of 3.0 L/min; drying gas flow of 10.0 L/min; interface voltage of 4.5 kV; desolation line temperature of 250 °C; heat block temperature of 400 °C; collision energy (CE) of −25 eV for MS/MS. Both nebulizing gas and drying gas were nitrogen, and the collision gas was argon. Precursor ion was set to m/z 249 [M + H]^+^ for MS/MS analysis.

The mobile phases A and B used to analyze the products of PipU, PipX and PipV, were 5 mM CH_3_COONH_4_/5 mM *N*,*N*-dimethylbutylamine (pH 7.5) and 100% acetonitrile. For other assessments, the mobile phases A and B were formic acid (0.1%) and 100% methanol, respectively. The LC flow rate was 1 mL min^−1^ and the column temperature was 40°C. The columns were equilibrated with 0.1% formic acid for 5 min followed by a linear gradient of 0%–100% methanol for 15 min, then 100% methanol for 5 min.

### Bioinformatic analyses

Amino acid sequences that were included in the homology alignment and construction of the PipM phylogenetic tree were determined using structurally characterized representatives of each subtype as queries (PDB codes: 1HZY, 1BF6, 1A4M, 1O12, 1M7J, 1ITQ, 1J5S, 4WHB [PuhB], 4WGX [MolA] and 3LS9).

The sequence alignments of PipM, U, V, W and X were using DNAMAN (Lynnon BioSoft; v. 7.0.2.176). The PipM phylogenetic tree was constructed using MAFFT (v. 7) and bootstrapped with 100 replicates.

### Structure comparison

The structure of PipM was predicted using AlphaFold3 with the amino acid sequence of PipM (copies: 4) and Zn^2+^ (copies: 4). PyMOL 3.0.4 was used in the structure comparison of PipM to PuhB (PDB code 4WHB [34]).

## Data availability

Nucleotide Sequence data that support the findings of this study have been deposited in DDBJ/GenBank database with the following accession codes: LC871763 for pipM, LC871764 for pipU, LC871765 for pipV, LC871766 for pipW, and LC871767 for pipX. The genomic and transcriptomic data in this study have been deposited in BioProject (Accession numbers: PRJDB37793).

## Supporting information

This article contains supporting information.

## Conflict of interest

The authors declare that they do not have any conflicts of interest with the content of this article.
