# Supplementary material for: A new amidohydrolase and β-oxidation–like pathway for piperine catabolism in soil actinomycetes
Source: J Biol Chem. 2025 Nov 5;301(12):110908. doi: 10.1016/j.jbc.2025.110908 (PMC12753220; doi:10.1016/j.jbc.2025.110908)
Supplement: Supporting information [file mmc1.pdf]

Supporting Information

A new amidohydrolase and β-oxidation–like pathway for piperine catabolism in soil actinomycetes

Pu Jian[1], Takuto Kumano[1,2,3#], Mio Kimura[1], Makoto Kurisaki[1], Yoshiteru Hashimoto[1,2,3], Michihiko Kobayashi[1,2,3,4#]

[1] Graduate School of Life and Environmental Sciences, University of Tsukuba, 1-1-1 Tennodai, Tsukuba, Ibaraki 305-8572, Japan

[2] Microbiology Research Center for Sustainability, University of Tsukuba, 1-1-1 Tennodai, Tsukuba, Ibaraki 305-8572, Japan

[3] Tsukuba Institute for Advanced Research (TIAR), University of Tsukuba, 1-1-1 Tennodai, Tsukuba, Ibaraki, 305-8577, Japan

[4] Center for Quantum and Information Life Sciences, University of Tsukuba, Tsukuba, Ibaraki, Japan

#Co-corresponding authors:
Michihiko Kobayashi, Ph.D. (Professor)
E-mail: kobayashi.m.fe@u.tsukuba.ac.jp

Takuto Kumano, Ph.D.
E-mail: kumano.takuto.fu@u.tsukuba.ac.jp

Supplementary Figures

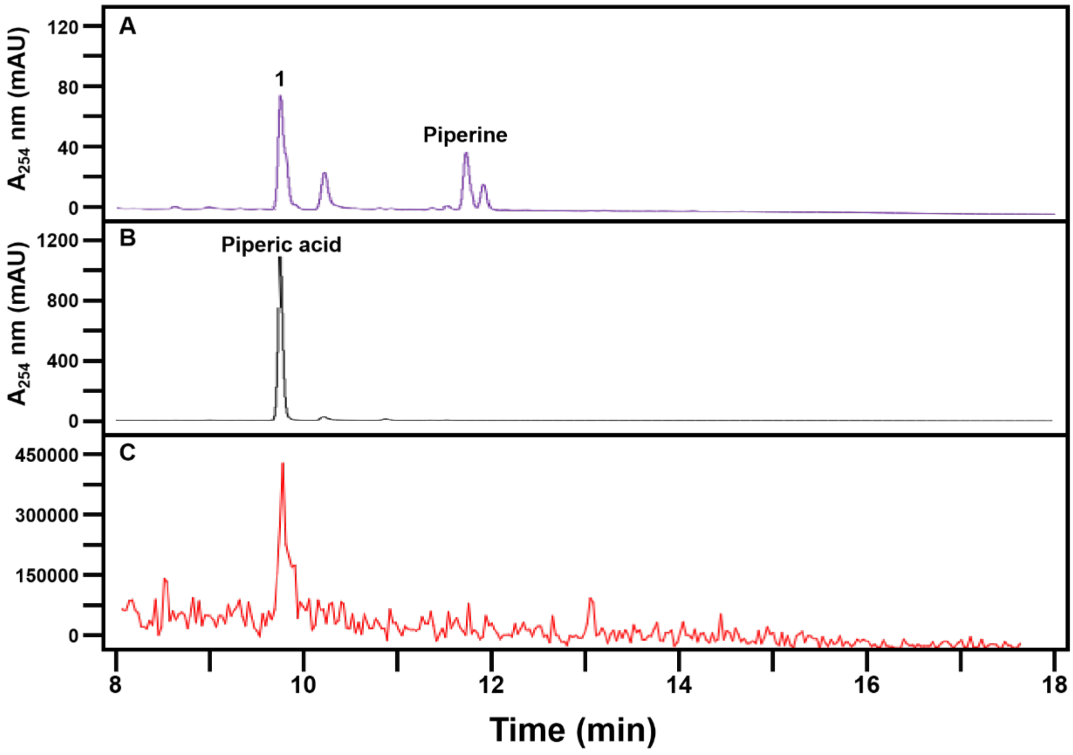

Supplementary Fig. 1. Determination of reaction products of piperine incubated with *R. ruber* No. 14 culture supernatants.

A, HPLC chromatogram of piperine incubated with culture supernatants.

B, HPLC chromatogram of authentic piperic acid.

C, LC-MS chromatogram of reaction mixture containing piperine and culture supernatants after 3 h incubation at m/z 219 in positive mode.

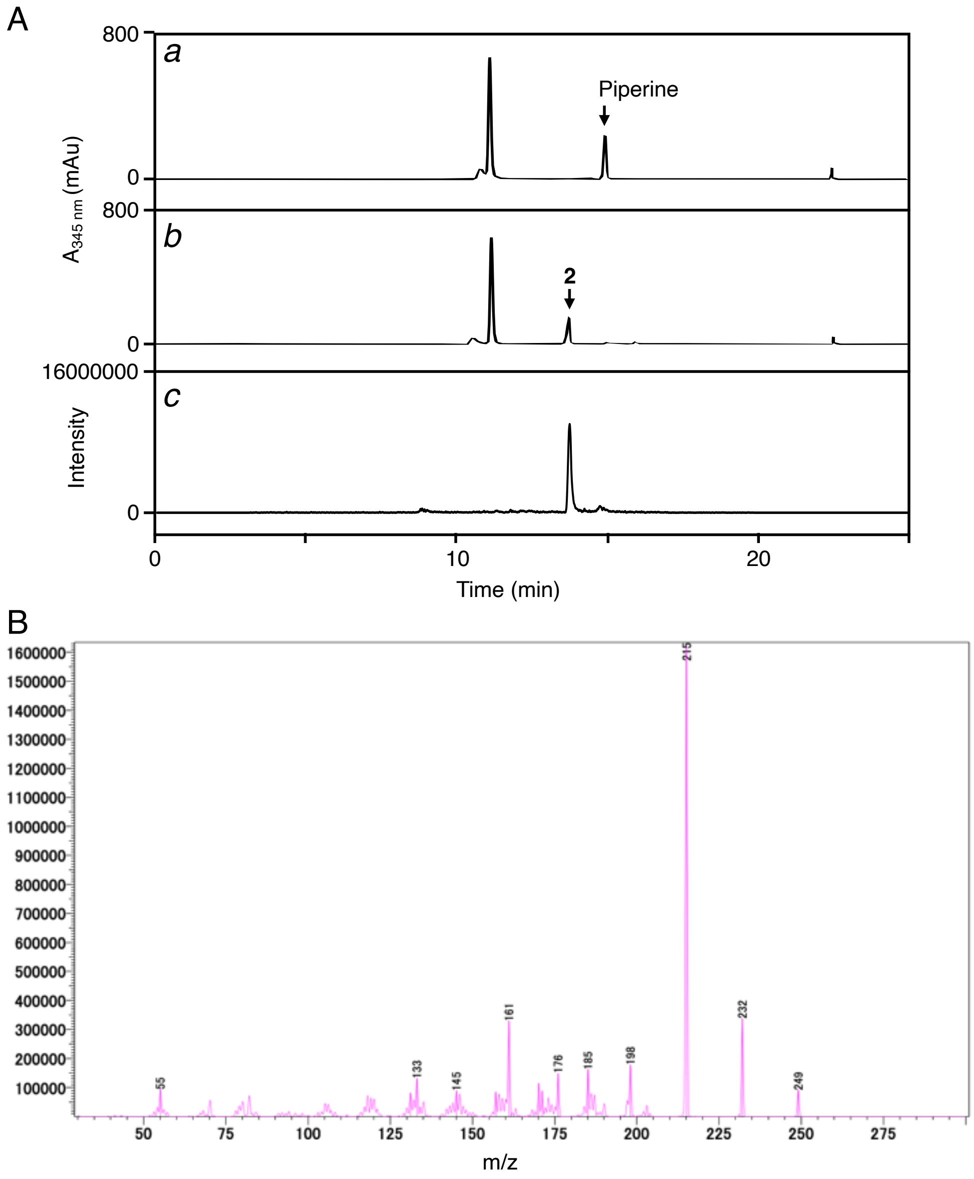

Supplementary Fig. 2. Identification of piperidine.

LC-MS/MS analysis of NBD-F added reaction mixtures of piperine and *R. ruber* No. 14 culture supernatants.

A, HPLC chromatograms of reaction mixtures at ***a***, 0 min and ***b***, after incubation for 18 h. **2**, Piperidine labeled with NBD-F. ***c***, LC-MS chromatogram of the reaction mixture at m/z 249 in positive mode.

B, MS/MS spectrum of peak **2**.

A

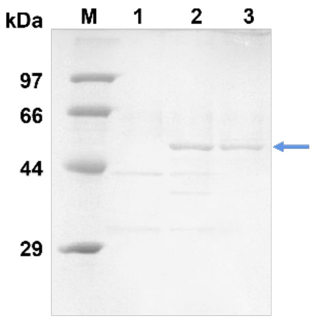

B

Step	Total protein (mg)	Total activity (μmol·min⁻¹)	Specific activity (μmol·min⁻¹·mg⁻¹)	Yield (%)	Fold
Culture supernatant	0.0164	7.32	0.0371	100	1
DEAE Sepharose	0.0285	4.67	0.386	63.7	10.4
HiPrep™ Butyl	0.0195	0.370	0.632	5.06	17.0
Resource™ Q	0.0425	0.380	4.47	5.19	120
BioAssist Q	0.0530	0.0175	3.29	0.724	88.7

C

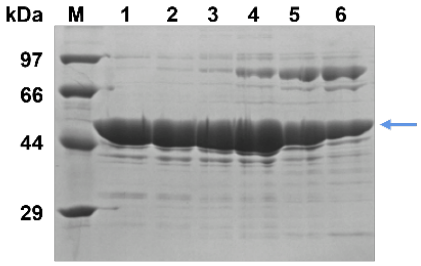

D

Step	Total protein (mg)	Total activity (μmol·min⁻¹)	Specific activity (μmol·min⁻¹·mg⁻¹)	Yield (%)	Fold
Cell-free extract	327	15.4	0.0472	100	1
HiPrep™ DEAE FF	119	14.9	0.125	96.8	2.64
Resource™ Q	58.4	14.0	0.240	90.9	5.08
Butyl 650M	21.8	5.49	0.253	35.6	5.36

Supplementary Fig. 3. Purification of PipM.

A, SDS-PAGE for purification of *R. ruber* No. 14 culture supernatants. PipM was purified as a single band on SDS-PAGE after four-step purification. M, molecular mass markers. numbers 1, 2, 3 represent column chromatography fractions. Blue arrow indicates PipM band.

B, Four-step purification of PipM from culture supernatants.

C, SDS-PAGE for purification of recombinant PipM. The enzyme was purified as a single band on SDS-PAGE after three-step purification. M, molecular mass markers. Numbers 1–6, column chromatography fractions. Blue arrow, PipM.

D, Three-step purification of recombinant PipM.

```
1       ATGTCTAGTTTTGCGTTATCGAATGTCACGCTGATCGACGGGCGCGGCGGTAGCCCAGCT   60
1       M  S  S  F  A  L  S  N  V  T  L  I  D  G  R  G  G  S  P  A     20

61      GAGGGCATGACGGTTGTTGTCGAGAGTGGCTCGATCACTCGCATCGTACCGACGTCCCAG   120
21      E  G  M  T  V  V  V  E  S  G  S  I  T  R  I  V  P  T  S  Q     40

121     TACGACGCGGGAGCCGGGCCAAAGGAAATTGATGCGGCTGGAAAATGGCTACTGCCCGGC   180
41      Y  D  A  G  A  G  P  K  E  I  D  A  A  G  K  W  L  L  P  G     60

181     TATATCAACGGCAACATCCACCTTCTTGACGGGATCATGATGATGGGCGTCGGCGGGGTC   240
61      Y  I  N  G  N  I  H  L  L  D  G  I  M  M  M  G  V  G  G  V     80

241     GAATACCTCGCCCGGTACGAGGGTTCCTTCTCTAAAGTCATCGAAGAGGGAGCGCAGATC   300
81      E  Y  L  A  R  Y  E  G  S  F  S  K  V  I  E  E  G  A  Q  I     100

301     ACCCTCAAGAATGGCGTGACGACAGTCTTCGACACCTGGGACGCTCGCGATCCAGTCCTG   360
101     T  L  K  N  G  V  T  T  V  F  D  T  W  D  A  R  D  P  V  L     120

361     GAAGCTCGGGACAGAATCAACGCTGGAACCGTCATCGGCTCCCGTATCTTCGCGGCAGGC   420
121     E  A  R  D  R  I  N  A  G  T  V  I  G  S  R  I  F  A  A  G     140

421     AACATAGTCGGGCTGGGTGGCCCATTCAGTCCTGACTTCAATTTCACTGCACGGCAGTCG   480
141     N  I  V  G  L  G  G  P  F  S  P  D  F  N  F  T  A  R  Q  S     160

481     ATATCGCAGAGCTTTGCCAACAGGATGGACCTGCTCTTTACTGCAGGGGTCGGAGCCGAA   540
161     I  S  Q  S  F  A  N  R  M  D  L  L  F  T  A  G  V  G  A  E     180

541     CTGACGTTGTTGCAAGAGCGCGAATTCCGGGCTCGGTTCAAGGACTACGTGCAGTCCGGG   600
181     L  T  L  L  Q  E  R  E  F  R  A  R  F  K  D  Y  V  Q  S  G     200

601     GTCGACATGGTCAAGATCGCCATCAGCGACCATCTCACGGCCCATCTCAACCCCGCTGCC   660
201     V  D  M  V  K  I  A  I  S  D  H  L  T  A  H  L  N  P  A  A     220

661     CTGCGGACCTATCATACCTTCCCGGAGAAGTGGGTCCGCATGATGGCAGAAGACGTCCAT   720
221     L  R  T  Y  H  T  F  P  E  K  W  V  R  M  M  A  E  D  V  H     240

721     TCAGCCGGACTGCCGTTCCTGTCGCACACGATGGCGGTGCCAGCCCTTGAGCTAGCGGCG   780
241     S  A  G  L  P  F  L  S  H  T  M  A  V  P  A  L  E  L  A  A     260

781     GATGTTGATGTCGATGTCATGATTCACCCGACATGGACATTCAACCAGGTAATCCCGGAA   840
261     D  V  D  V  D  V  M  I  H  P  T  W  T  F  N  Q  V  I  P  E     280

841     GAGTTGGTCAATCGAATCGCGGAACGTCGAATTGGTGTGGGAATACAGCCCATCACTGAC   900
281     E  L  V  N  R  I  A  E  R  R  I  G  V  G  I  Q  P  I  T  D     300

901     GACTACGCGGATCGGCTTCTCGTCCACAGAAATCCATTCGGAACACTGAATAGCCCCGAA   960
301     D  Y  A  D  R  L  L  V  H  R  N  P  F  G  T  L  N  S  P  E     320

961     CACCAGAAGAACGAACGAAACTTCATCGAAGCCGGCGCCAATGTGATGGTTGCTACCGAC   1020
321     H  Q  K  N  E  R  N  F  I  E  A  G  A  N  V  M  V  A  T  D     340

1021    GCCGGTTGTACGTCACACGACGTCATGCAGGACCTTGGACCAGACCTGCAGGAGGGCAGG   1080
341     A  G  C  T  S  H  D  V  M  Q  D  L  G  P  D  L  Q  E  G  R     360

1081    CCCTGGTCTTTGGGGGCTGACCACTTCACGTGGGTCAAAGCGTTGCGCACACGAGGCCTC   1140
361     P  W  S  L  G  A  D  H  F  T  W  V  K  A  L  R  T  R  G  L     380

1141    ACCACGATGGGAGCGATTCAAGCCCTCACCCACAATGTGGCGGAGGCATACAACAAGCTA   1200
381     T  T  M  G  A  I  Q  A  L  T  H  N  V  A  E  A  Y  N  K  L     400

1201    GACACCATCGGCACTGTCGAGGTGGGAAAGCTAGCCGATCTCGTGCTGCTGAACTCGGAT   1260
401     D  T  I  G  T  V  E  V  G  K  L  A  D  L  V  L  L  N  S  D     420

1261    CCACTTGTTGACAGCGAGAACCTGAGCGACATCGCAGCAATCTATAAGGAAGGTGTCCTA   1320
421     P  L  V  D  S  E  N  L  S  D  I  A  A  I  Y  K  E  G  V  L     440

1321    GTTGACCGGTCGGCATTGCCGAACCCCCAGATCGTTACGGCGCCAACGGGCACGCCTGTT   1380
441     V  D  R  S  A  L  P  N  P  Q  I  V  T  A  P  T  G  T  P  V     460

1381    CCGAACTGA   1389
461     P  N  *     462
```

Supplementary Fig. 4. Nucleotides and deduced amino acid sequences of *pipM*.

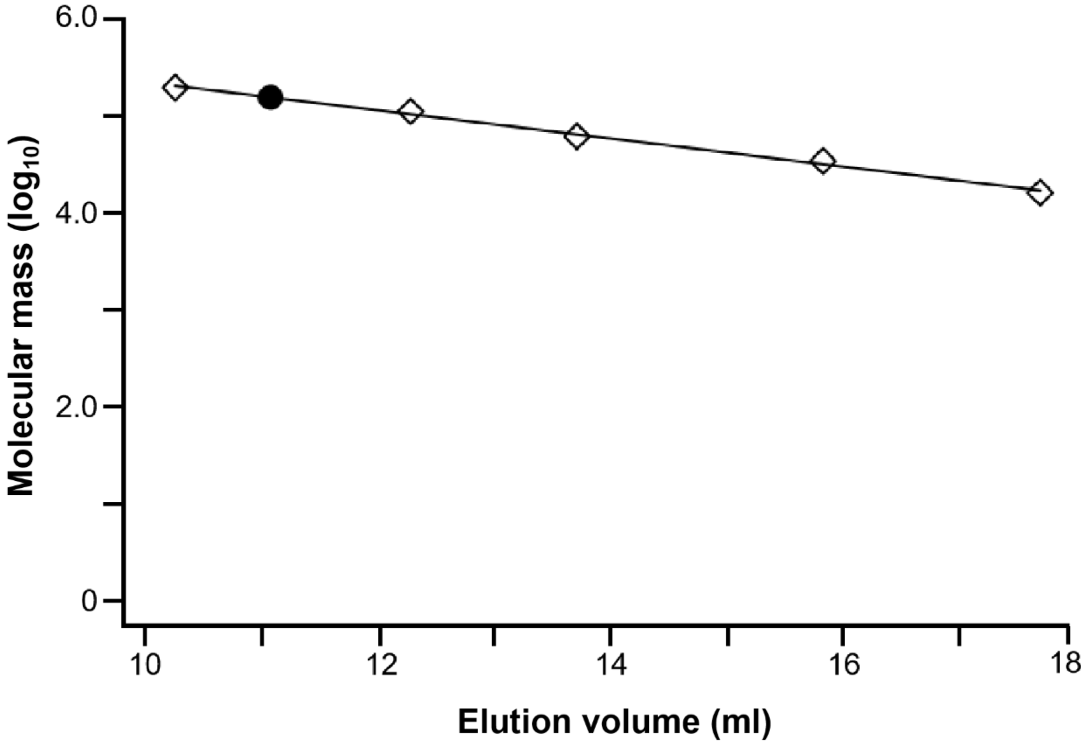

Supplementary Fig. 5. Estimation of native molecular mass of PipM using gel-filtration column chromatography.

Estimation of molecular mass of native PipM. ◊, standard proteins in Log_{10} form; ●, PipM. Standard proteins: cytochrome *c*, 12.4 kDa, 17.5 mL; myokinase, 32 kDa, 15.8 mL; enolase, 67 kDa, 13.8 mL; lactate dehydrogenase, 142 kDa, 12.5 mL; glutamate dehydrogenase, 290 kDa, 10.6 mL. Molecular masses of standard proteins revealed linear relative relationship to elution volume: $y = -0.1902x + 7.4888$; $R^2 = 0.9956$. Native PipM eluted at 11.3 mL, indicating a molecular mass of ~ 220 kDa.

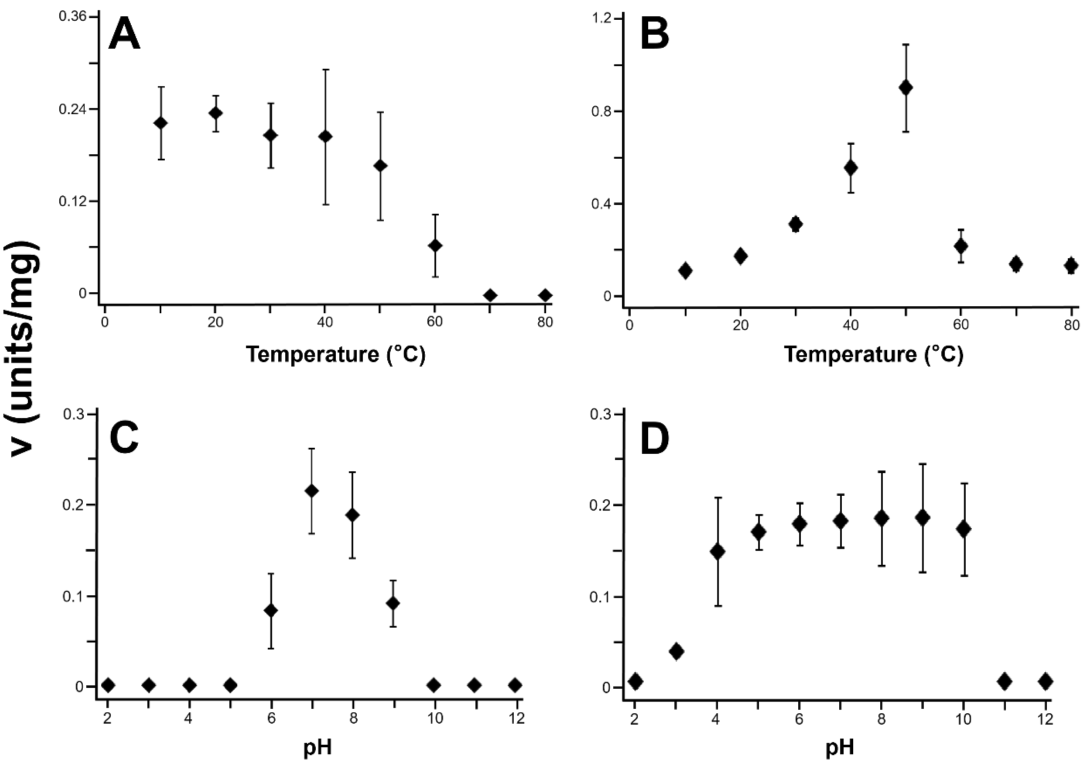

Supplementary Fig. 6. Temperature and pH effects on reaction activity of PipM.

A, Temperature stability.
B, Temperature dependency.
C, pH dependency.
D, pH stability.
Error bars, standard deviation of three independent experiments.

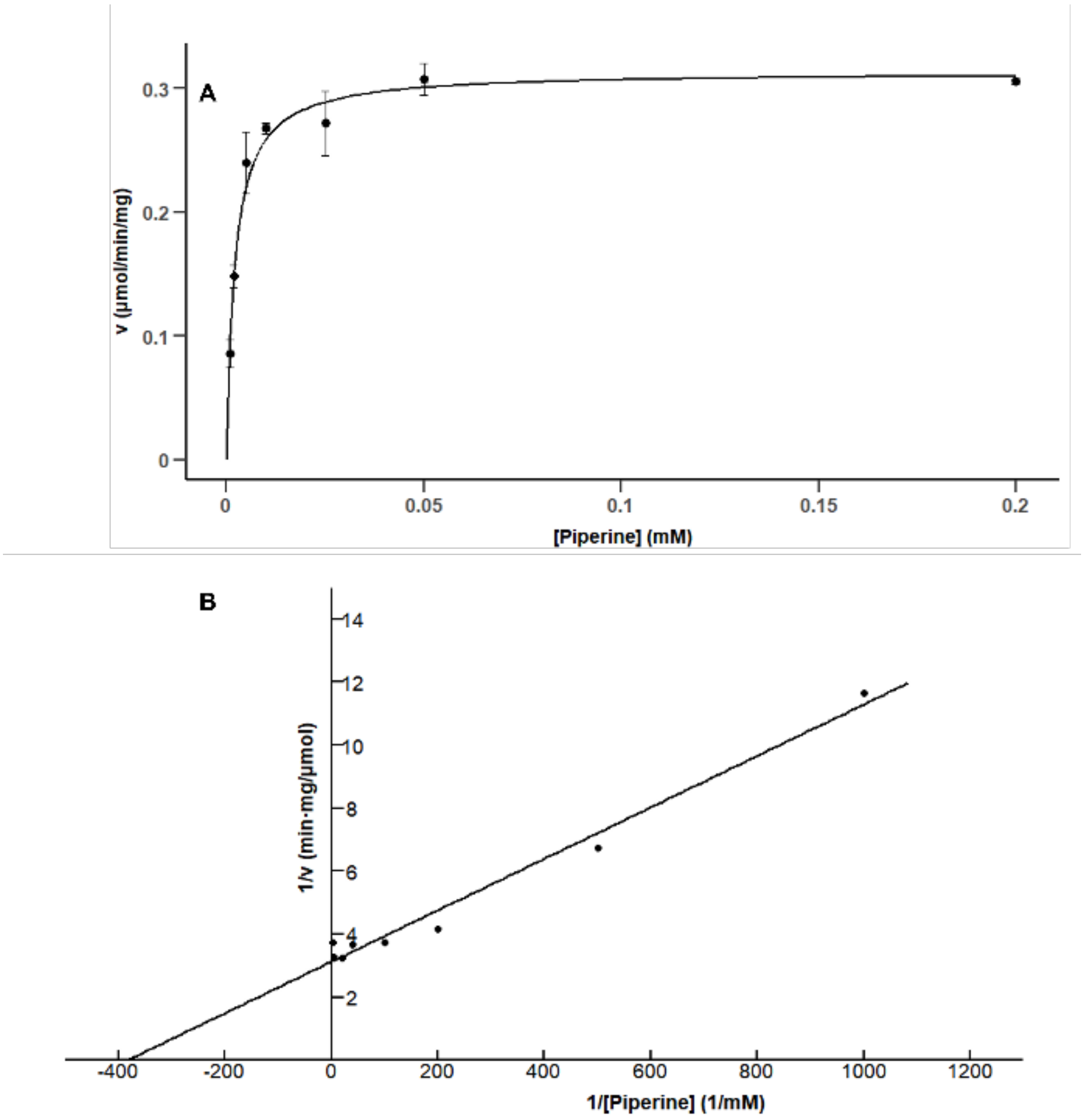

Supplementary Fig. 7. **Kinetic parameter analysis of PipM.**

A, Michaelis-Menten plot of PipM. Black points, average velocity of piperic acid formation. Error bars, standard deviation.

B, Lineweaver-Burk plot of PipM. Black points represent the reciprocal of the average velocity of piperic acid formation. Experiments were performed three times, respectively.

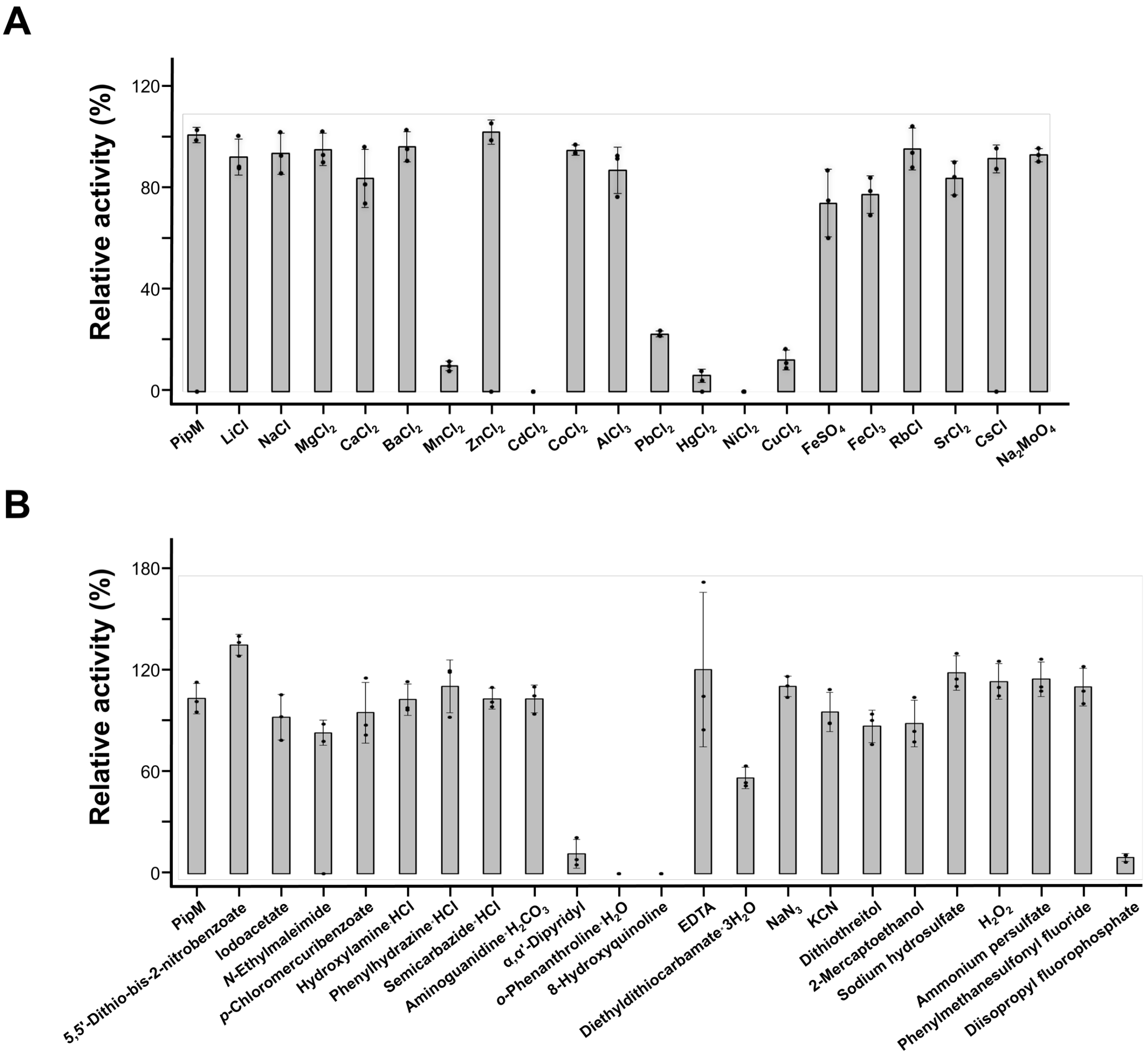

Supplementary Fig. 8. Inhibitor analysis of PipM.

Effects of **A,** metal inhibitors and **B,** non-metal inhibitors on PipM. Metal ions Mn^{2+}, Cd^{2+}, Pb^{2+}, Hg^{2+}, Ni^{2+}, and Cu^{2+} obviously inhibited PipM activity. Chelating agents α,α'-dipyridyl, *o*-phenanthroline·H_2O and 8-hydroxyquinoline, and serine-modifying agent diisopropyl fluorophosphate inhibited PipM activity. All experiments proceeded in triplicate.

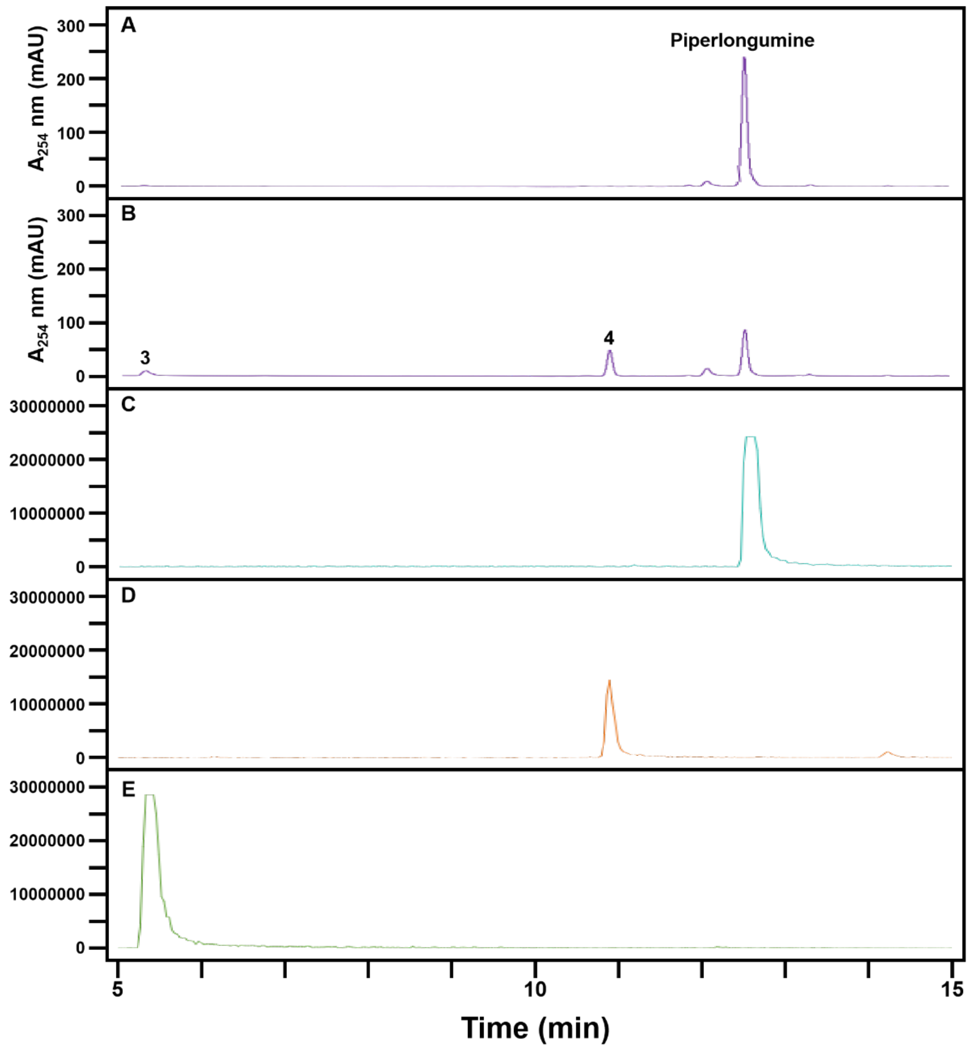

Supplementary Fig. 9. Reaction of piperlongumine with PipM.

LC-MS analysis of the reaction mixtures of piperlongumine and *R. ruber* No. 14 culture supernatants. **A and B,** HPLC chromatograms of reaction mixtures at 0 min and after incubation for 2 h, respectively (**3** and **4,** degradation products of piperlongumine). **C–D**, LC-MS chromatograms of reaction mixture after incubation for 2 h at m/z 318, 239, and 98 in positive mode.

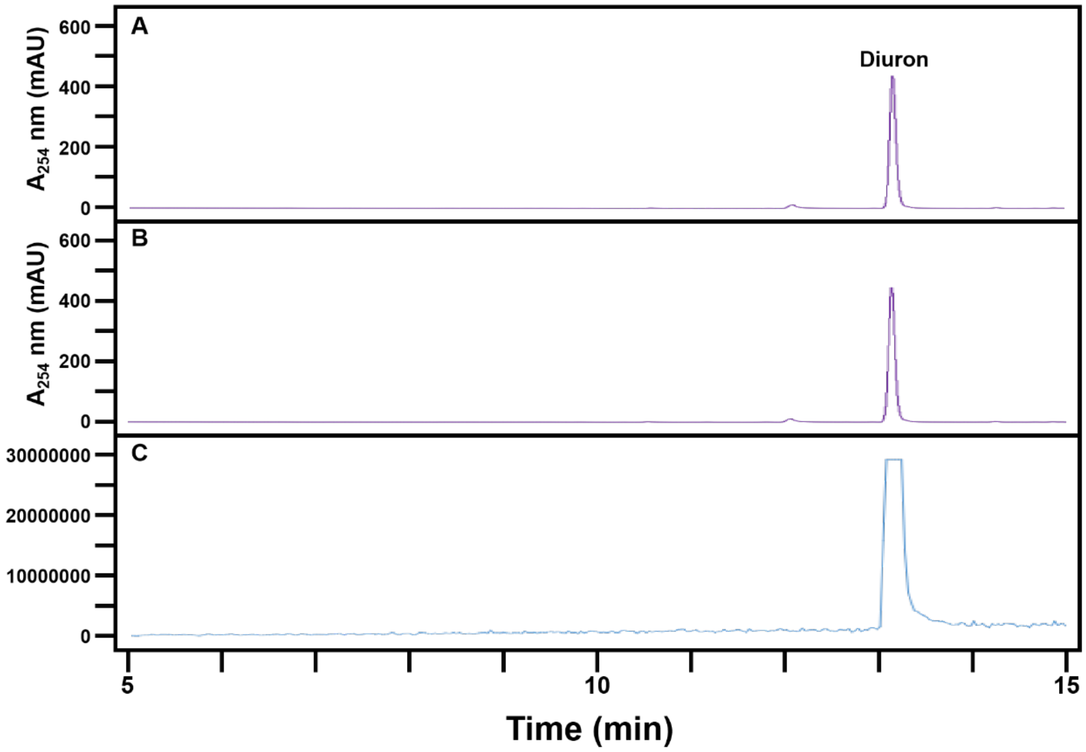

Supplementary Fig. 10. **Reaction of diuron with PipM.**

LC-MS analysis of the reaction mixtures of diuron and *R. ruber* No. 14 culture supernatants. **A and B**, HPLC chromatograms of reaction mixtures at 0 min and after incubation for 2 h, respectively. **C**, LC-MS chromatogram of reaction mixture after 2 h incubation at m/z 233 in positive mode.

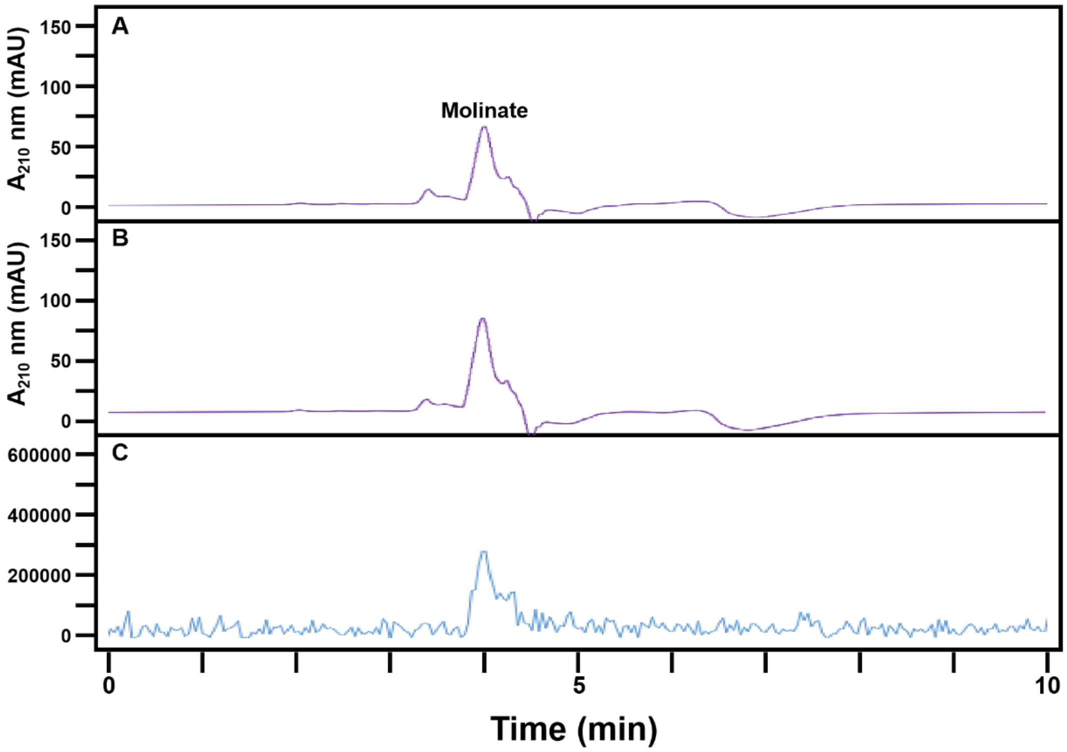

Supplementary Fig. 11. Reactions between molinate and PipM.

LC-MS analysis of the reaction mixtures of molinate and *R. ruber* No. 14 culture supernatants. **A and B**, HPLC chromatograms of reaction mixtures at 0 min and after incubation for 2 h. **C**, LC-MS chromatogram of reaction mixture after incubation for 2 hat m/z 188 in positive mode.

```
1     GTGATCTATCGACCCCGAACCGTCGAACTGGACGTACCATCCGTATCTCTGTCCGAGTAC   60
1     V  I  Y  R  P  R  T  V  E  L  D  V  P  S  V  S  L  S  E  Y     20

61    GTCCTGCGGGATGCCGATAAGCGCGGAGAAAAGATCGCGCTGGTGGATGGTGTGACTGGC   120
21    V  L  R  D  A  D  K  R  G  E  K  I  A  L  V  D  G  V  T  G     40

121   CGCTCGATCAGCTATGCCCAACTCGACGCGCTCTCCGCACGCGGTGCGGCCGGTCTCGCT   180
41    R  S  I  S  Y  A  Q  L  D  A  L  S  A  R  G  A  A  G  L  A     60

181   GCGCACGGAATCCAGCCCGGCGACGTGGTTGCGCTCGCCAGCCATAACTGCCCGGACTTC   240
61    A  H  G  I  Q  P  G  D  V  V  A  L  A  S  H  N  C  P  D  F     80

241   GCGGTTGCGGTCTACGCGATTCTGCGGGCGGGAGCAACGGTGACACTGCTCAACCCGATT   300
81    A  V  A  V  Y  A  I  L  R  A  G  A  T  V  T  L  L  N  P  I     100

301   CTCACGGTTCACGAAATGACCAAGCAGCTGGCGCACTCCGGAACGAAAGTGGTCATCTGC   360
101   L  T  V  H  E  M  T  K  Q  L  A  H  S  G  T  K  V  V  I  C     120

361   ACACCCGAGATCGCACAGAAGGTGGCGGCGGCCTGCGATGAGACCGGAGTTCGGTCCCAC   420
121   T  P  E  I  A  Q  K  V  A  A  A  C  D  E  T  G  V  R  S  H     140

421   TTCGTGATCGGCGAGGACACGGGATCAGGCGCGTTCGAATCGCTGCTTAAGTATGAGCCG   480
141   F  V  I  G  E  D  T  G  S  G  A  F  E  S  L  L  K  Y  E  P     160

481   CGCACCGCGATCCCTGGATTGAACCCCGCCACCGCGCTGGCCGCTCTGCCCTACTCGAGC   540
161   R  T  A  I  P  G  L  N  P  A  T  A  L  A  A  L  P  Y  S  S     180

541   GGAACTACCGGAATGAGCAAGGGGGTCATGCTGACCCACCGTAACCTCGTGGCGAACCTC   600
181   G  T  T  G  M  S  K  G  V  M  L  T  H  R  N  L  V  A  N  L     200

601   GCGCAGGTCAGATCCGCGTGGAATCTCGATGAGACGGACGTGGTGTGCGCGGCGTTACCC   660
201   A  Q  V  R  S  A  W  N  L  D  E  T  D  V  V  C  A  A  L  P     220

661   TTCTTCCACATCTACGGGTTCACCATCATCCTCAACTCGACGTTGGTTGCCGGCGGCACG   720
221   F  F  H  I  Y  G  F  T  I  I  L  N  S  T  L  V  A  G  G  T     240

721   ATCGTGACCCTTCCCCAGTTCGACCTTCGCACCTACCTCGGCGTCGTCGAGCAGTACGGG   780
241   I  V  T  L  P  Q  F  D  L  R  T  Y  L  G  V  V  E  Q  Y  G     260

781   GTGACGTTCGGCCACCTCGCGCCCCCGCTCGTCCTGGCGCTGGCGGCGGCACCAGAAGTC   840
261   V  T  F  G  H  L  A  P  P  L  V  L  A  L  A  A  A  P  E  V     280

841   GACGACTACGACCTGTCCTCGATGCGTAAGGCCGTGTCGGGCGCTGCGCCCCTAGACGAG   900
281   D  D  Y  D  L  S  S  M  R  K  A  V  S  G  A  A  P  L  D  E     300

901   GACGCGGTCGCGCGCGCAGAAGCCCGCACCGGCATCGTCATCCGGCAGGGCTACGGCATG   960
301   D  A  V  A  R  A  E  A  R  T  G  I  V  I  R  Q  G  Y  G  M     320

961   ACCGAGGCTAGCCCCGGAACACACTGCGTTCACATCGACGATTTCGAGCGCACCCCCTCG   1020
321   T  E  A  S  P  G  T  H  C  V  H  I  D  D  F  E  R  T  P  S     340

1021  GGTTCGGTCGGTCGGCTACTTCCTGCCACCGAAGCGCGGATTGTCGACCCGGTCACGGAG   1080
341   G  S  V  G  R  L  L  P  A  T  E  A  R  I  V  D  P  V  T  E     360

1081  GACGACGCCCCTCTCGGTCAACGAGGCGAATTATGGGTCCGCGGACCGCAGGTCATGGCC   1140
361   D  D  A  P  L  G  Q  R  G  E  L  W  V  R  G  P  Q  V  M  A     380

1141  GGCTACCTCGACAACGCCGACGCTACTACCACAACCATCACTGATGGCTGGCTGCGCACC   1200
381   G  Y  L  D  N  A  D  A  T  T  T  T  I  T  D  G  W  L  R  T     400

1201  GGCGACATCGCTGTGGTCGACGGCGAAGACTTCTTTATCGTCGACCGCCTCAAGGAACTG   1260
401   G  D  I  A  V  V  D  G  E  D  F  F  I  V  D  R  L  K  E  L     420

1261  ATCAAGTACAAGGGCTACCAAATCGCACCTGCCGAACTCGAGGCCGTACTGCTGAACCAC   1320
421   I  K  Y  K  G  Y  Q  I  A  P  A  E  L  E  A  V  L  L  N  H     440

1321  CACCTGGTGAGCGACGCCGCCGTCATAGGCATACCTCACCCTTCCGGAGGGGAAGCACCA   1380
441   H  L  V  S  D  A  A  V  I  G  I  P  H  P  S  G  G  E  A  P     460

1381  AAAGCGTTCGTAGTCTCCACCGGCGACCTATCAGCCGATGAGCTCATCGCGTGGGTGAGC   1440
461   K  A  F  V  V  S  T  G  D  L  S  A  D  E  L  I  A  W  V  S     480

1441  GAGAGGGTAGCCCCCTATAAGAAGGTCCGAGCCGTCGCCTTCGTCGATCAGATCCCGAAG   1500
481   E  R  V  A  P  Y  K  K  V  R  A  V  A  F  V  D  Q  I  P  K     500

1501  TCACCGGCGGGGAAAATCCTGCGGCGCGTTCTCAAGGAAGACTCCGCGCAGCAGAACGTG   1560
501   S  P  A  G  K  I  L  R  R  V  L  K  E  D  S  A  Q  Q  N  V     520

1561  GTCGAATCCGCATAA   1575
521   V  E  S  A  *     524
```

Supplementary Fig. 12. Nucleotides and deduced amino acid sequences of *pipU*.

```
1      ATGGATTTGAACAACAAGGTCGCAATCGTCACCGGGAGCGGACAGGGACTGGGCCTGGCT   60
1      M  D  L  N  N  K  V  A  I  V  T  G  S  G  Q  G  L  G  L  A     20

61     TATGCGCAGGACCTCGCCCGCCACGGTGCCGCAGTGGTGATCAACGATGTCAACCAAGAC   120
21     Y  A  Q  D  L  A  R  H  G  A  A  V  V  I  N  D  V  N  Q  D     40

121    ACCGCGAACAACGCAGTGGCGTCGATCCTCGACGAGGGTGGCCGTGCCGTCGCTGTCGTA   180
41     T  A  N  N  A  V  A  S  I  L  D  E  G  G  R  A  V  A  V  V     60

181    GCGCCAGTCGGGTCCACTGATACCGCGCAAGAGCTTGTCGCCACCGCAGTCGAGAACTTC   240
61     A  P  V  G  S  T  D  T  A  Q  E  L  V  A  T  A  V  E  N  F     80

241    GGTCGCCTCGACGTCATGGTCACGAACGCAGGCATCTTGCGCGACACTGTGTTATGGAAG   300
81     G  R  L  D  V  M  V  T  N  A  G  I  L  R  D  T  V  L  W  K     100

301    ATGAGCGACGACGACTTCGACGCCGTCATCAACGTCCATCTACGCGGCACGTTCACCTGT   360
101    M  S  D  D  D  F  D  A  V  I  N  V  H  L  R  G  T  F  T  C     120

361    GTCCGTGAGGCCGCGATCCGCTTGCGTGAACAGGGAGAGGGCGGCCGCATCATCGCCATC   420
121    V  R  E  A  A  I  R  L  R  E  Q  G  E  G  G  R  I  I  A  I     140

421    GGATCCCCCGCCGGGCAACGCGGCAACTTCGGGCAAACCAACTACGCTGCCGCGAAGGCA   480
141    G  S  P  A  G  Q  R  G  N  F  G  Q  T  N  Y  A  A  A  K  A     160

481    GGCATCGTTGGTATGGTCCGCACCTGGGCGATGGAGTTGCAACGCGATGGTGTCACTGTC   540
161    G  I  V  G  M  V  R  T  W  A  M  E  L  Q  R  D  G  V  T  V     180

541    AACGCCGTGTGCCCCGTCGCCGCGACGGCGATGACCGAGACCATTCCCTTCCTTGCCCCG   600
181    N  A  V  C  P  V  A  A  T  A  M  T  E  T  I  P  F  L  A  P     200

601    CACATCGCGTCAATGAAGAACGGCGATCCGCTTCCGCCGATCATTCGCCGTGACGTCGGC   660
201    H  I  A  S  M  K  N  G  D  P  L  P  P  I  I  R  R  D  V  G     220

661    ATGGGCACGCCGCACGATGCCGCCGGAATCATCTCCTTCCTGGCGTCTGACGATGCCGCC   720
221    M  G  T  P  H  D  A  A  G  I  I  S  F  L  A  S  D  D  A  A     240

721    GGCATCACTGGTCAGGCGTTCTCGGTCGGCGGCGACCGCCTCGCCTTGTGGTCTCACCCC   780
241    G  I  T  G  Q  A  F  S  V  G  G  D  R  L  A  L  W  S  H  P     260

781    GTCCTTACCGCCACTGCGTTCCACGACGGAGGATGGAGCGCAGACGATATCTCTGCTCAG   840
261    V  L  T  A  T  A  F  H  D  G  G  W  S  A  D  D  I  S  A  Q     280

841    TGGGGTTCCGCCTTTGCCGCCTCCGTCGAATCCGTCGGTGAGAAGTTCCCGGACGAGCTG   900
281    W  G  S  A  F  A  A  S  V  E  S  V  G  E  K  F  P  D  E  L     300

901    ATGTCACGATGA   912
301    M  S  R  *     303
```

Supplementary Fig. 13. Nucleotides and deduced amino acid sequences of *pipV*.

```
1     ATGACGAAGTACGAGTACGGAATCGACTTCGCGGCAATCGAGGCCGTCGACTTCCACACT   60
1     M  T  K  Y  E  Y  G  I  D  F  A  A  I  E  A  V  D  F  H  T     20

61    CACGTTCAGCTGGACGGGTGTGGTAGGCACGCGATGGACGAGGTCCTCCTCGCGGCGACG   120
21    H  V  Q  L  D  G  C  G  R  H  A  M  D  E  V  L  L  A  A  T     40

121   GAGAAATACTTCAAGTCCCCCGAGGACCAGAACCCTACGGTCGACGACGTCGCCGAACAC   180
41    E  K  Y  F  K  S  P  E  D  Q  N  P  T  V  D  D  V  A  E  H     60

181   TACCGCGCCCGCAACATGGCTGCGGTGGTCTTTACAGTCGACGCTGCCTCTGCCACCGGA   240
61    Y  R  A  R  N  M  A  A  V  V  F  T  V  D  A  A  S  A  T  G     80

241   CACCAGGCGAACTCCGTTGAGGAGATTGCCGAGGGCGCAGCCCGCCACAACGACGTCCTC   300
81    H  Q  A  N  S  V  E  E  I  A  E  G  A  A  R  H  N  D  V  L     100

301   ATCCCCTTCGGCTCCGTCGACCCATTGCAAGGCAAAGCGGCCGTCAAGAAGCTCCGACGC   360
101   I  P  F  G  S  V  D  P  L  Q  G  K  A  A  V  K  K  L  R  R     120

361   CTCATCGACAACCACGGCATCAAGGGCGTCAAGTTTCACCCGAGCATGCAGGGGTTCGCG   420
121   L  I  D  N  H  G  I  K  G  V  K  F  H  P  S  M  Q  G  F  A     140

421   CCTAACGATCATGCCCACTACCCGCTCTACGACGCGATAGCCGAAGCCGGTATCCCCGCG   480
141   P  N  D  H  A  H  Y  P  L  Y  D  A  I  A  E  A  G  I  P  A     160

481   CTCTTCCACACTGGGCAGACTGGCATCGGGGCCGGACTGCCCGGCGGACATGGCATCAAG   540
161   L  F  H  T  G  Q  T  G  I  G  A  G  L  P  G  G  H  G  I  K     180

541   CTTCGCTATTCGGACCCGATGCTTCTCGACGACGTCGCGGCCGACTTCCCCGATCTGACA   600
181   L  R  Y  S  D  P  M  L  L  D  D  V  A  A  D  F  P  D  L  T     200

601   ATCGTTATGGCCCACCCCTCCGTGCCGTGGGTGGACGCCCAGATCTCTGTCGCCACTCAC   660
201   I  V  M  A  H  P  S  V  P  W  V  D  A  Q  I  S  V  A  T  H     220

661   AAGGCCAATGTCTACATCGACCTCTCGGGTTGGTCCCCGAAGTACTTCCCGCCGCAGCTC   720
221   K  A  N  V  Y  I  D  L  S  G  W  S  P  K  Y  F  P  P  Q  L     240

721   GTGCGCGCCGCGAACACCACGCTGCGCGACAAGGTCCTCTTCGGCTCCGACTTCCCGGTT   780
241   V  R  A  A  N  T  T  L  R  D  K  V  L  F  G  S  D  F  P  V     260

781   ATCCAGGTCGATCGATGGATGGCAGACTTCGCCCAACTCGACATCAAAACTGAGGTGGCC   840
261   I  Q  V  D  R  W  M  A  D  F  A  Q  L  D  I  K  T  E  V  A     280

841   CCAAAGATATTCAAGGGCAACGCGCTTCGCGTTCTAGGTATCGCAGAATGA   891
281   P  K  I  F  K  G  N  A  L  R  V  L  G  I  A  E  *     296
```

Supplementary Fig. 14. Nucleotides and deduced amino acid sequences of *pipW*.

```
1     ATGACCACCTCCGCCCCTGCAGGCACCACCGTCCAGACGCCAAGCGAACTCCTCACCCTC   60
1     M  T  T  S  A  P  A  G  T  T  V  Q  T  P  S  E  L  L  T  L     20

61    GAAGGGAAGGCCCTCGGCACATCCACATGGCGCGAGGTTACCCAATCCGATGTAGATATG   120
21    E  G  K  A  L  G  T  S  T  W  R  E  V  T  Q  S  D  V  D  M     40

121   TTCGCCAAGGCAACCGGCGACGAGCAATGGATCCACGTCGACGTCGAACGCGCGAAGACC   180
41    F  A  K  A  T  G  D  E  Q  W  I  H  V  D  V  E  R  A  K  T     60

181   GGCCCCTTCGGGAGCACCATCGCACACGGTTACATGACCCTATCGCTGATTGCCCCCCTG   240
61    G  P  F  G  S  T  I  A  H  G  Y  M  T  L  S  L  I  A  P  L     80

241   TTCGACGAGGTTCTCACCATCACAGATCTCGGCATGGGCGTCAACTACGGACTGAACAAG   300
81    F  D  E  V  L  T  I  T  D  L  G  M  G  V  N  Y  G  L  N  K     100

301   GCTCGGTTCCCCGCACCTCTCCCCGTCGGCTCCCGCGTCCGCCTCACTGCCAGCGTGACA   360
101   A  R  F  P  A  P  L  P  V  G  S  R  V  R  L  T  A  S  V  T     120

361   GGCGCGGAAGAAATCCGGGGCGGCGTACAGATCATCGTCGCCATCACTATCGAACGCGAC   420
121   G  A  E  E  I  R  G  G  V  Q  I  I  V  A  I  T  I  E  R  D     140

421   GGTGGCGACAAGCCCGTCTGTTTCGCCGAAGCAGTTCTGCGCTACTACAACTGA   474
141   G  G  D  K  P  V  C  F  A  E  A  V  L  R  Y  Y  N  *     157
```

Supplementary Fig. 15. **Nucleotides and deduced amino acid sequences of *pipX*.**

```
PipU       .......................VIYRPRTVELDVPSVSLSEYVLRDADKRGEKIALVDG     37
CouL       ..................................MDNEGIGSWLERRITMTPKNEALVF.     25
Atu1416    MTTTPREVSFWTPRLTVDRRADGTILLGQEEPLGAYPDHMGQRFARWARETPDRIWMAEK     60

PipU       V..TGRSISYAQLDALSARGAAGLAAHGIQPGDVVALASHNCPDFAVAVYAILRAGATVT     95
CouL       ...DGRAVTYEEMALRTRRLAHGLHALGVEKGDCVGFFGFNDPAALEVMFAAGLLGATYL     82
Atu1416    SGDGWATLSYAKAWASIRAIGEALARRGLTQDTPVLILSGNSIAHALMALGAQHVGVPSA    120

PipU       LLNPILTVH....EMTKQLAHS.GTKVVICT...PEIAQKVAAACDETGVRSHFVIGEDT    147
CouL       PLNARLTAE....EARYVLGDSRCTTVIFGD...QQADVAQELAQSDTPVTTWIGLGASW    135
Atu1416    ALAPAYALTGGDYAKLRDIAAQISPGLIFADDAAPYADAIATVFGAEVPVVSLTGTVEGR    180

PipU       GSGAFESLLKY...EPRTAIPGLNPATALAALPYSSGTTGMSKGVMLTHRNLVAN..LAQ    202
CouL       STHTYEGVHAG...QPDTRIDEQVGLDDLSVLMYSSGTTGAPKGVMLSHGNMLWN..ALN    190
Atu1416    ETLSFDALTATHLTDAEATAAAAVTPDTVAKFLFTSGTTGSPKAVIQTQRMLCSNQQMVQ    240

PipU       VRSAWNLDETDVVCAALPFFHIYGFTIILNSTLVAGGTIVTLP....QFDLRTYLGVVEQ    258
CouL       QLLAQDMTSKERTLSVAPLFHIGGIGGAVTPTLLNGGTVVLLR....KFDAGVVLDTIEK    246
Atu1416    QCFTFLKETPPVVVDWAPWNHTASGNKVFNMVIYNGGTYYIDDGRPTPKAIHTTIETLRD    300

PipU       YGVTFGHLAPP....LVLALAAAPEVDDYDLSSMRKAVSGAAPLDEDAVARAE......A    308
CouL       ERITTFFAVPT....MIQELWHHPRFADADLSSLRAICVAGAPLPEALISPWQ......D    296
Atu1416    ISPTWYFNVPLGYQMLLDAFETDTKLRDTFFKRIQMLFYAGAGMSQPVWDRLTRVCDEMV    360

PipU       RTGIVIRQGYGMTEASPGTHCVHIDDFERTPSGSVGRLLPATEARIVDPVTEDDAPLGQR    368
CouL       RD.VAITQAYGLTETAPSVTMLSSADVR.SKIGSAGKRTFFTDVDVVRPDGSSAEPH.EI    353
Atu1416    PGGVLLTSGFGATETGP...FSVTNTARQSKSGNLGLPAPGVTLKLV........PQGDK    409

PipU       GELWVRGPQVMAGYLDNADATTTTITDGWLRTGDIAVVDGE......DFFIVDRLKELIK    422
CouL       GEIVAKGPNVMLGYLNQPEATARTIVDGWLHTGDAGYFDDEG.....FLFICDRYKDMYI    408
Atu1416    MEARVKSPSITPGWKNAALTQDAFDEEGFYRFGDAFRFADPDDPSQGLLFDGRLAENFKL    469

PipU       YKGYQIAPAELEAVLLNHHL..VSDAAVIGIPHPSGGE...APKAFVVSTG..DLSADEL    475
CouL       SGGENVYPAEVEAALLRLEG..IREAAVIGVPHEKWGE...TGMAFVVAADGTTLDEETV    463
Atu1416    ASGTWVAVGPLRAKLTDDLGGLASDVVIAGEGHTELGALVVPNRAALREIAGEDIEGEEL    529

PipU       IAWVSERVAPYKKVRAVAFVDQIPKSPAGKILRRVLKEDSAQQNVVESA...........    524
CouL       RARLREKLAGFKIPTFIQIAEALPRTATGKIRKPDLRKLAASRPVSTS............    511
Atu1416    LGHPEVRRRTAERLAAHAKAATGSASRVMRMMYMSVPLDFDKGEVTDKGSINQRAVLRHR    589

PipU       ...................                                             524
CouL       ...................                                             511
Atu1416    ADLVDSLWSEDPRVIHILK                                             608
```

Supplementary Fig. 16. **Sequence alignment of PipU with homologues in *R. jostii* RHA1 and *A. fabrum* sp.**

CouL is from *R. jostii* RHA1 (15) and Atu 1416 is from *A. fabrum* sp. (16). Pink and blue highlights indicate identical and similar residues, respectively. **Supplementary Fig. 20** shows functions of each enzyme.

```
PipV       MDLNNKVAIVTGSGQGLGLAYAQDLARHGAAVVINDVNQDTANNAVASILDEGGRAVAVV    60
CouN       MDLSNKVAVVTGSGQGLGLAYAKDLVRHGAAVVINDVNQATADAAVAEITAAGGRAVAVV    60
Atu1415    MKIAGQVAIVTGGASGLGAATARRLAAEGAKVGILDFDGDGAAAMAREIG.....GMAVK    55

PipV       APVGSTDTAQELVATAVENFGRLDVMVTNAGI.LRDTVLWK...MSDDDFDAVINVHLRG   116
CouN       APVGSTETAQKLVAGAIDAFGRLDVMVTNAGI.LRDKVLWK...MTDDDFDAVINVHLRG   116
Atu1415    TDVGLEASVADAVAEVKQRLGAPRIAVSCAGIGLAGRVVGRDGALSTDLFEKTIRVNLMG   115

PipV       TFTCVREAAIRLR.....EQGEGGRIIAIGSPAGQRGNFGQTNYAAAKAGIVGMVRTWAM   171
CouN       TFTCVREAVLKFR.....EQGDGGRIICIGSPAGQRGNFGQTNYSGAKSGIVGMVRTWAM   171
Atu1415    TYYVMSHAAREMMALEPLESGERGVVVNTASVAYEDGQIGQVAYSASKGAIASMCLPAAR   175

PipV       ELQRDGVTVNAVCP.VAATAMTETIPFLAPHIASMKNGDPLPPIIRRDVGMGTPHDAAGI   230
CouN       ELQRAGITANAVCP.VAATAMTETVPFLAPYIEGMKNGEPLPDIIRRDVGLGTPEDAAGI   230
Atu1415    EMAKQGVRVMAIAPGLFNTPMMEGLPQET..VDGIVANVPFP......HRLGDPAEYAQL   227

PipV       ISFLASDDAAGITGQAFSVGGDRLALWSHPVLTATAFHDGGWSADDISAQWGSAFAASVE   290
CouN       ISFLASDAAAEITGQAFAVGGDRLALWSHPDLTAVEYHDGGWSADDIAAQWTGTFGDAVQ   290
Atu1415    VCQIL..DSPYLNGSVIRLDG.AVRLPQR...............................   253

PipV       SVGEKFPDELMS                                                   302
CouN       SVGEEFPEELMA                                                   302
Atu1415    ............                                                   253
```

Supplementary Fig. 17. Sequence alignment of PipV with homologues in *R. jostii* RHA1 and *A. fabrum* sp.

CouN is from *R. jostii* RHA1 (15) and Atu 1415 is from *A. fabrum* sp. (16). Pink and blue highlights indicate identical and similar residues, respectively. **Supplementary Fig. 20** shows functions of each enzyme.

```
PipW      MTKYEYGIDFAAIEAVDFHTHVQLDGCG.RHAMDEVLLAATEKYFKSPEDQN...PTVDD    56
CouO      MSRYEYGIDFDKIDAIDIHTHVEIDGCG.HRSLDDELMAASEKYFKSGEERT...PSIDA    56
Atu1421   .......MNIDELIAIDVHTHAEEPCCGPRDDGYDEFQAGMAKYFKNPAGHKGMLPTVQE    53

PipW      VAEHYRARNMAAVVFTVDAASATGHQANSVEEIAEGAARHNDVLIPFGSVDPLQGKAAVK   116
CouO      IADHYRARNMAAVVFTVDAASASGHPTNSVEEIAEGAARHNDVLIPFGSVDPWQGKAAVR   116
Atu1421   TAAYYRERKIGCVIFPVDAERETGFRRYENEEVAKIAAENSDIMIPFASIDPAKGKAGAR   113

PipW      KLRRLIDNHGIKGVKFHPSMQGFAPNDHAHYPLYDAIAEAGIPALFHTGQTGIGAGLPGG   176
CouO      RVHRLVDDYGVKGFKFHPSMQGFEPNDRQFYPLYEAITEAGVPALFHTGQTGIGAGLPGG   176
Atu1421   EARRLVREFGVKGFKFHPTMQGFYPNDRMAYPLYEAIAEEGAITLFHTGQTGVGAGLRGG   173

PipW      HGIKLRYSDPMLLDDVAADFPDLTIVMAHPSVPWVDAQISVATHKANVYIDLSGWSPKYF   236
CouO      HGIKLRYSDPMLLDDVAADFPDLTLIMAHPSVPWVDSQISIATHKANVFIDLSGWSPKYF   236
Atu1421   MAMRLKFSNPIHLDDVAVDFPDMPIILAHPSFPWQEEALSVATHKPNVYIDMSGWSPKYF   233

PipW      PPQLVRAANTTLRDKVLFGSDFPVIQVDRWMADFAQLDIKTEVAPKIFKGNALRVLGIA    295
CouO      PPQLVKAANSMLGGKVLFGSDFPVIQVDRWMKDFANLDIKPEVAPLIFKQNALRVLGIK    295
Atu1421   PPILVQYANSLLKHKMLFGSDWPAMTPERWLNDFANISIKDEVRPLILKENARRLLKL.    291
```

Supplementary Fig. 18. **Sequence alignment of PipW with homologues in *R. jostii* RHA1 and *A. fabrum* sp.**

CouO is from *R. jostii* RHA1 (15) and Atu 1421 is from *A. fabrum* sp. (16). Pink and blue highlights indicate identical and similar residues, respectively. **Supplementary Fig. 20** shows functions of each enzyme.

```
PipX      ..................................MTTSAPAGTTVQTPSELLTLEGKALG    26
CouM      ....................................MTTATTTRVATLADLAALEGQSLG    24
Atu1417   MDTTETLVTYELKGAVALIGLNRPDKRNAISDRFVEAIHEAVQRAQKEARAGVIFGHGKH    60

PipX      TSTWREVTQS.DVDMFAKATGDEQWIHVDVERAKTG.PFGSTIAHGYMT........LSL    76
CouM      TSSWIDIPQQ.RINTFADATDDHQWIHVDPERATAESPFGGPIAHGYLT........LSL    75
Atu1417   FCAGLDLAEHSEKPLFEAVKGSRRWHAVFDGIERGTIPFVAAISGAAVGGGFELAASTQV   120

PipX      IAPLFDEVLTITDLGMGVNYG....LNKARFPAPLPVGSRVRLTASVTGAEEIR.GGVQI   131
CouM      IIPMWDEVLTVDSVTMAVNYG....LNKVRFTNPVPAEGRVRLNATLQSVEELPKGGVQV   131
Atu1417   RVADQTAFFALPEGQRGIFVGGGGSVRIARLIGAARMGDMMLTGRALKATEAEAWGGVSY   180

PipX      IVAI..TIERDGGDKPVCFAEAVLRYYN................................   157
CouM      TVAG..QIELEGSERPAVVVEAVYRFFE................................   157
Atu1417   VVPEGEALTRAVALAEQMATNAEFTNYAIINALPRIADMSSEDGLFAESMVASLATMTDD   240

PipX      ....................                                           157
CouM      ....................                                           157
Atu1417   AKDRLRAFLEKRAAKVQDPN                                           260
```

Supplementary Fig. 19. **Sequence alignment of PipX with homologues in *R. jostii* RHA1 and *A. fabrum* sp.**

CouM is from *R. jostii* RHA1 (15) and Atu 1417 is from *A. fabrum* sp. (16). Pink and blue highlights indicate identical and similar residues, respectively. **Supplementary Fig. 20** shows functions of each enzyme. Although Atu1417 does not share significant sequence similarity with PipX and CouM, three enzymes have similar functions.

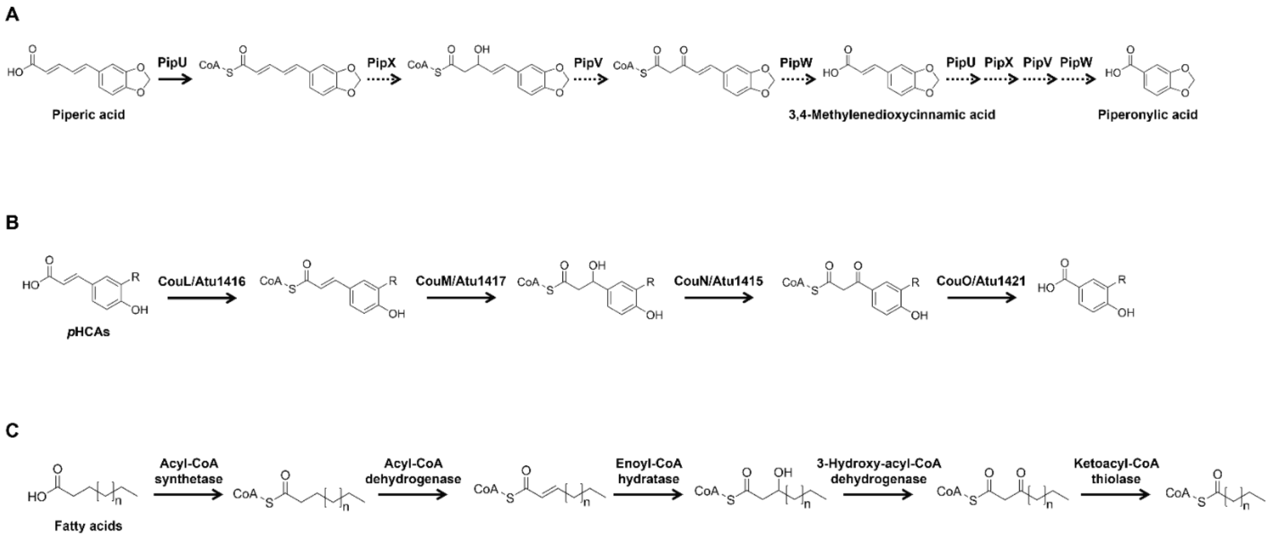

Supplementary Fig. 20. Comparison of β-oxidation among piperic acid, *p*HCAs and fatty acids metabolism.

β-oxidation-like pathways of **A**, piperic acid (dashed arrows: proposed reaction steps); **B**, *p*-hydroxycinnamates (*p*HCAs); and **C**, fatty acids.

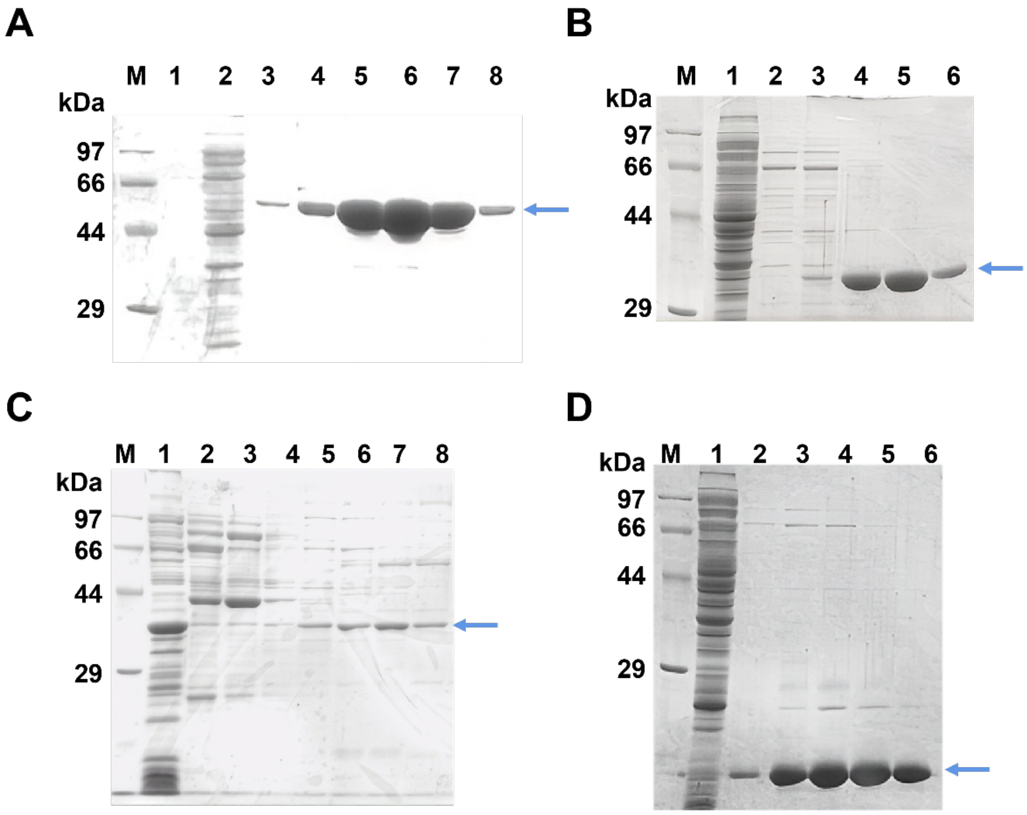

Supplementary Fig. 21. **The SDS-PAGE of the purified PipU, V, W and X.**

SDS-PAGE for purification of the recombinant PipU, V, W and X by column chromatography from *E. coli*. **A–D**, PipU, PipV, PipW, PipX, respectively. M, molecular mass markers. Numbers indicate column chromatography fractions. Blue arrows, purified enzymes.

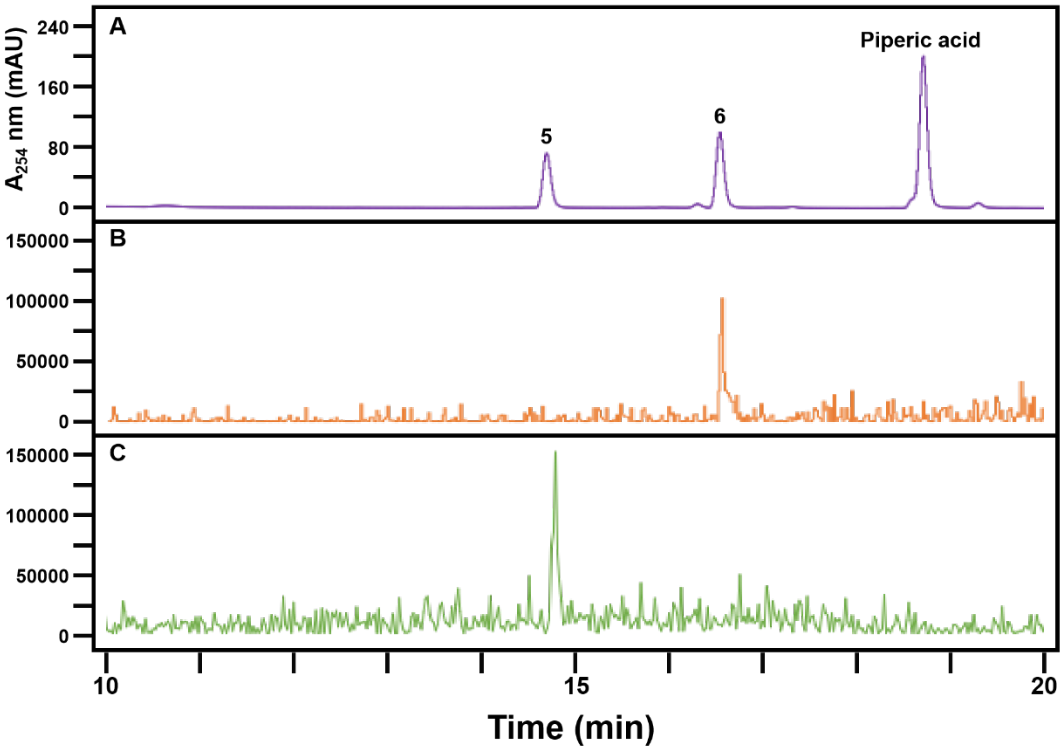

Supplementary Fig. 22. Molecular masses of products 5 and 6.

LC-MS analysis of the reaction mixture of piperic acid and purified Pip, V, W and X after incubation for 4 h. **A**, HPLC chromatogram of reaction mixture. **5** and **6**, Degradation products of piperic acid. **B and C**, LC-MS chromatograms of reaction mixture at m/z 191 and m/z 165, respectively, in negative mode.

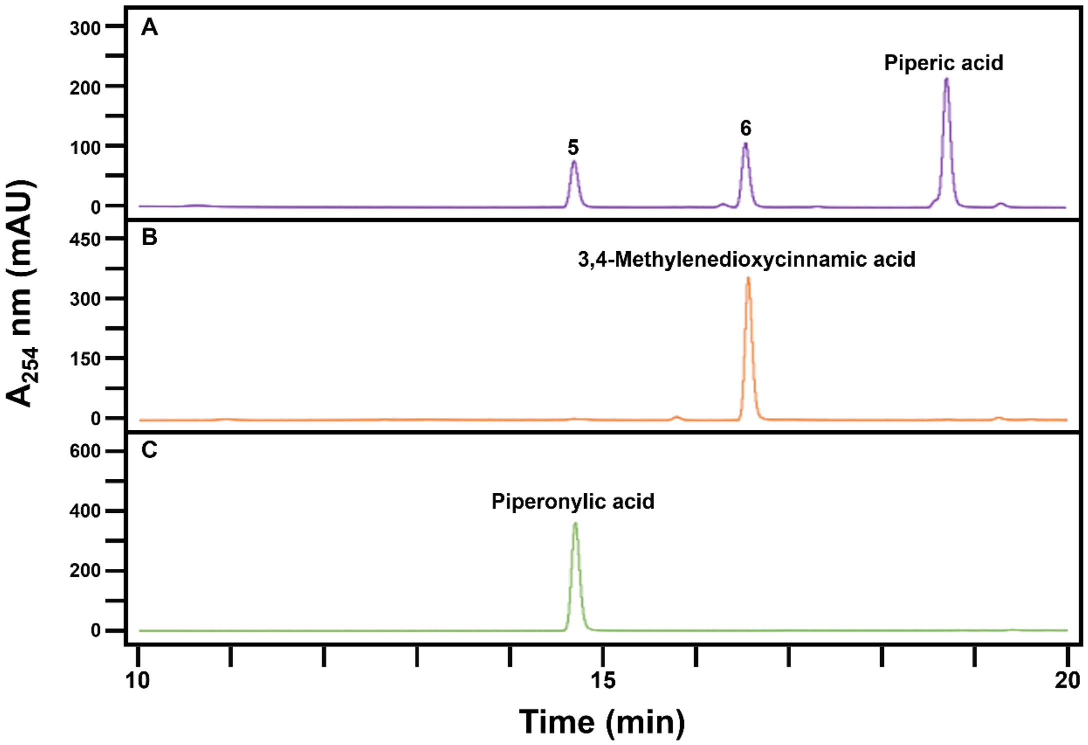

Supplementary Fig. 23. Comparison of authentic 3,4-methylenedioxycinnamic acid and piperonylic acid to products 5 and 6.

HPLC chromatograms of **A,** 4-h reactions between piperic and purified PipU, V, W and X; **B**, authentic 3,4-methylenedioxycinnamic acid; and **C**, authentic piperonylic acid. **5** and **6,** Degradation products of piperic acid.

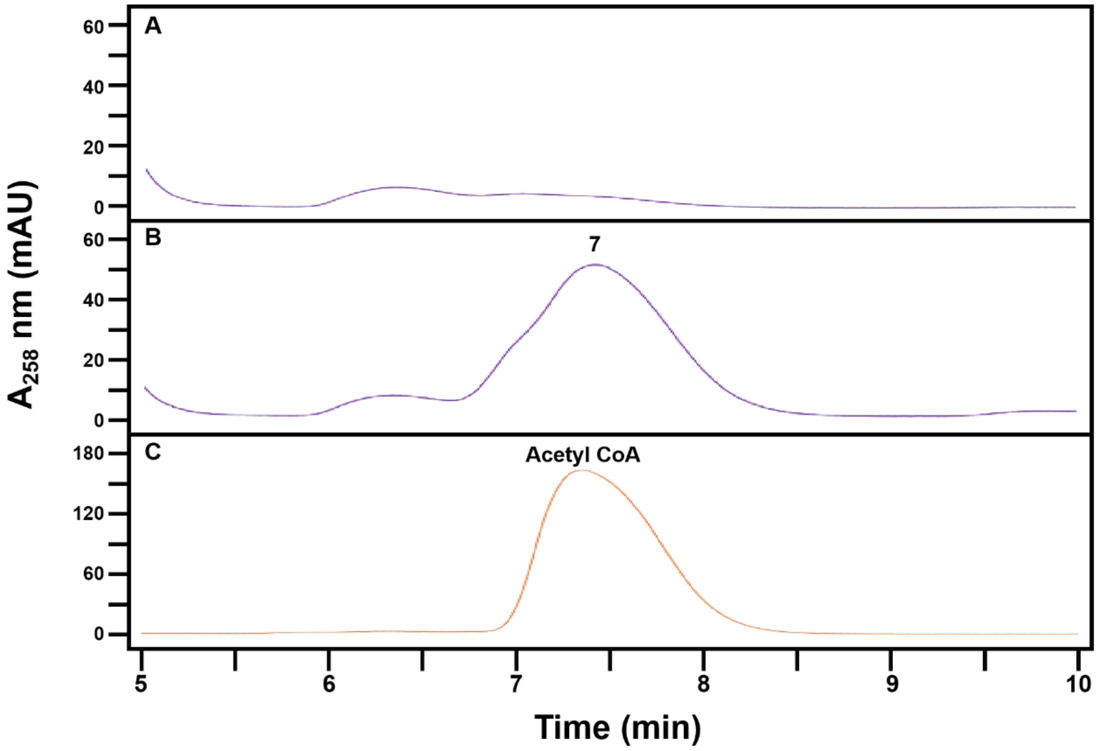

Supplementary Fig. 24. Identification of acetyl-CoA formation in the degradation of piperic acid.

LC-MS analysis of reaction mixtures of piperic acid and purified PipU, V, W and X. HPLC chromatograms of reaction mixtures at **A**, 0 min and **B**, after 18 h incubation, and of **C**, authentic acetyl-CoA. Product **7** was detected as single peak under conditions specific for detecting acetyl-CoA.

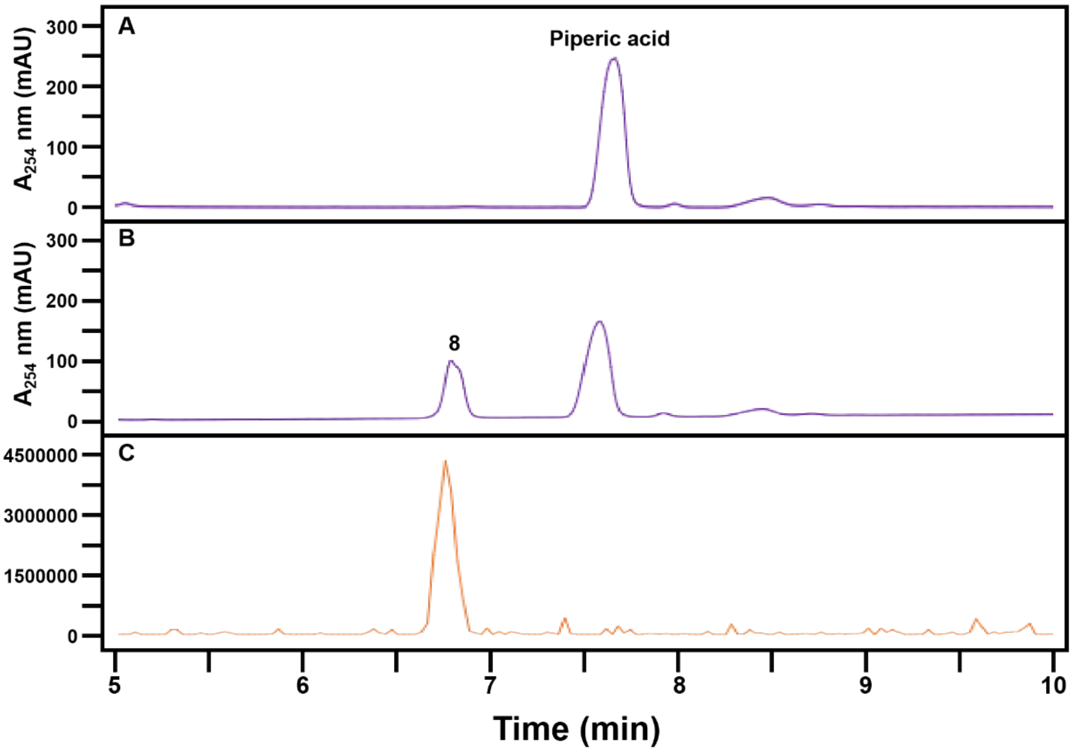

Supplementary Fig. 25. Molecular mass identification of the product of piperic acid with PipU.

LC-MS analysis of reaction mixtures of piperic acid and purified PipU. HPLC chromatograms of reaction mixtures at **A,** 0 min and **B**, after incubation for 2 h. **C**, LC-MS chromatogram of reaction after 2 h incubation at m/z 966 in negative mode. **8,** Product of piperic acid and PipU.

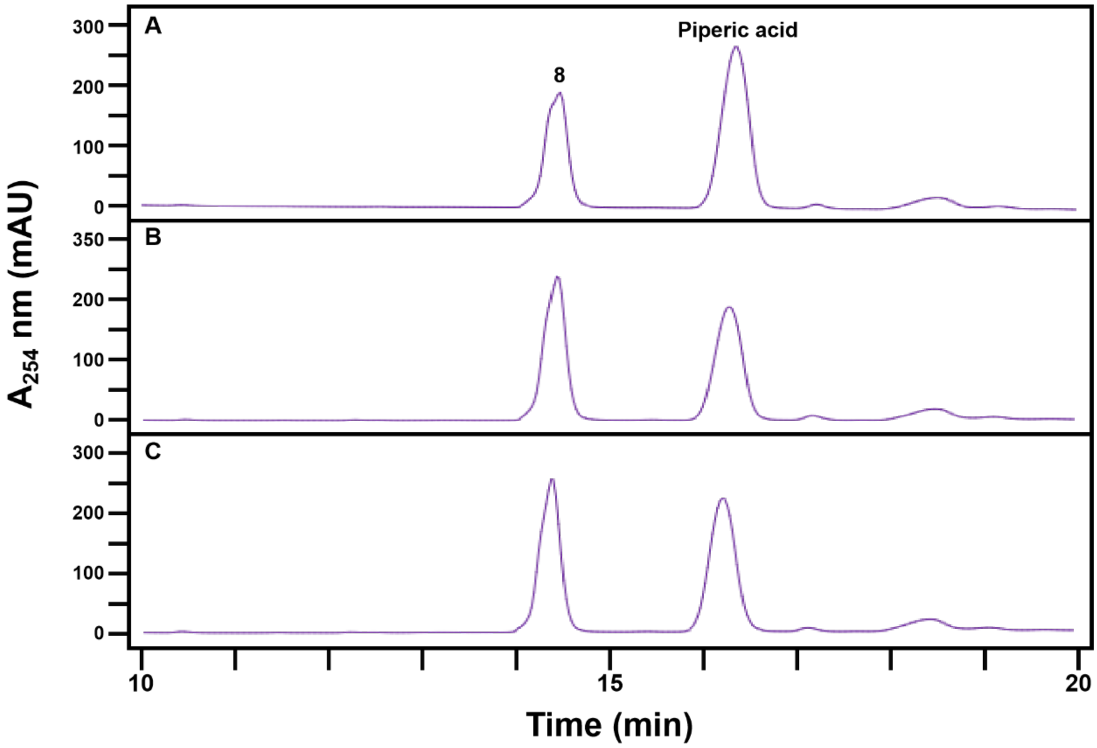

Supplementary Fig. 26. Identification of PipX and PipW products.

HPLC chromatograms of 4-h reactions between piperic acid and **A**, purified PipU; **B**, purified PipU and PipX; **C**, purified PipU, PipX, and PipV. **8,** CoA-conjugated piperic acid.

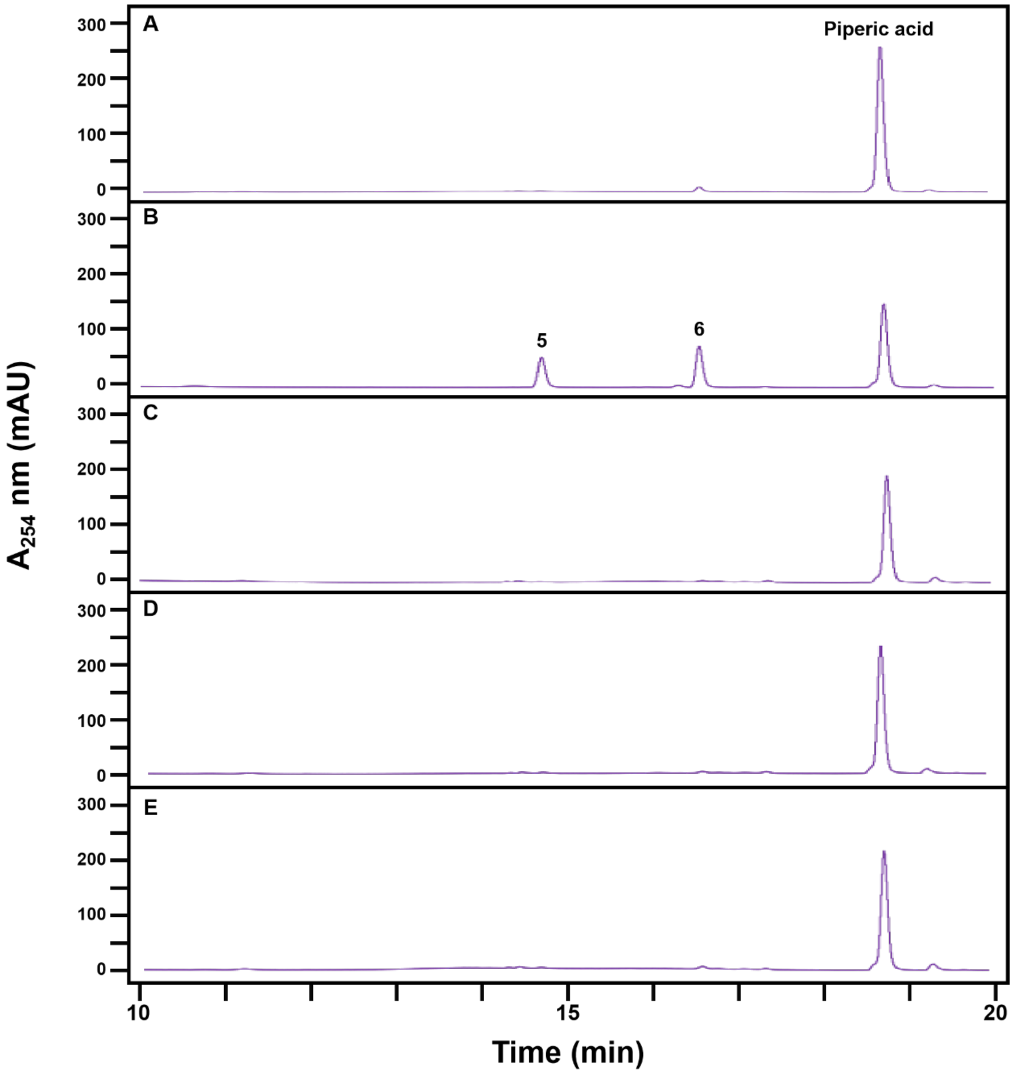

Supplementary Fig. 27.   Piperic acid metabolism requires PipU, V, and W.

HPLC chromatograms of reactions between piperic acid and **A**, purified PipU, V, W and X at 0 min; **B**, purified PipU, V, W and X at 4 h; **C**, purified PipU, W and X at 4 h, **D**; purified PipU, V and X at 4 h, **E**, purified PipU, V and W at 4 h. **5,** Piperonylic acid; **6**, 3,4-methylenedioxycinnamic acid.

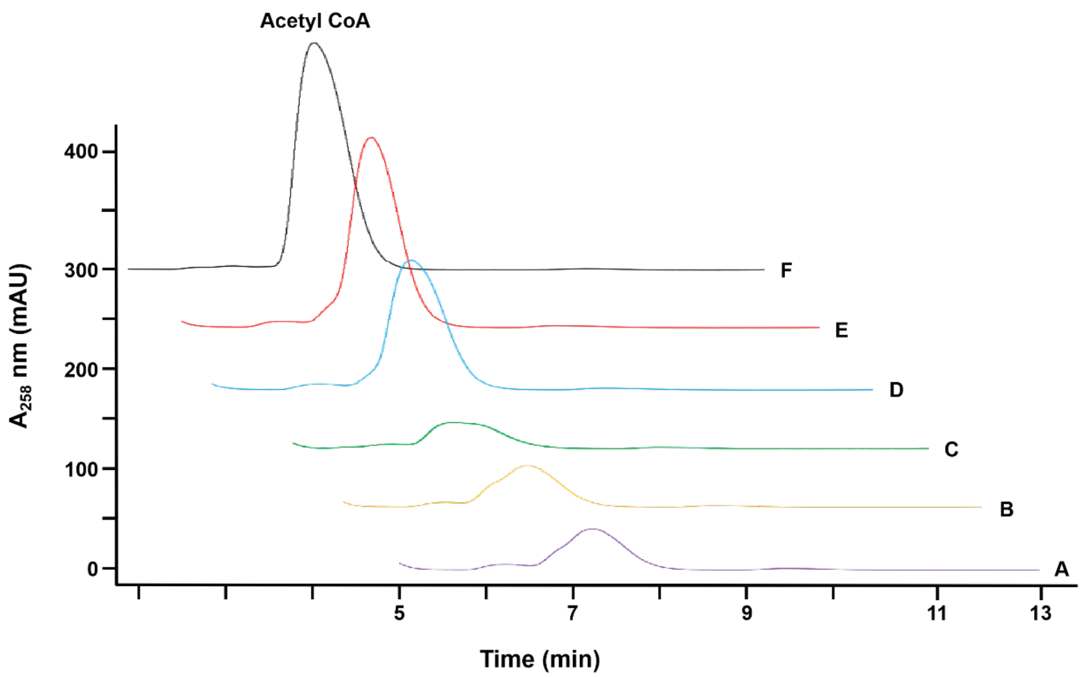

Supplementary Fig. 28. **Substrate availability of PipU, V, W and X.**

HPLC analysis of candidate substrates and purified PipU, V, W and X under specific conditions to detect acetyl-CoA. **A–E**, HPLC chromatograms of reaction mixture after 18 h of incubation between purified PipU, V, W and X **and A,** piperic acid; **B**, *trans*-2-dodecenoic acid; **C,** crotonic acid; **D,** 3,4-methylenedioxycinnamic acid; **E**, 4-hydroxy-3-methoxycinnamic acid. **F**, HPLC chromatogram of authentic acetyl-CoA.

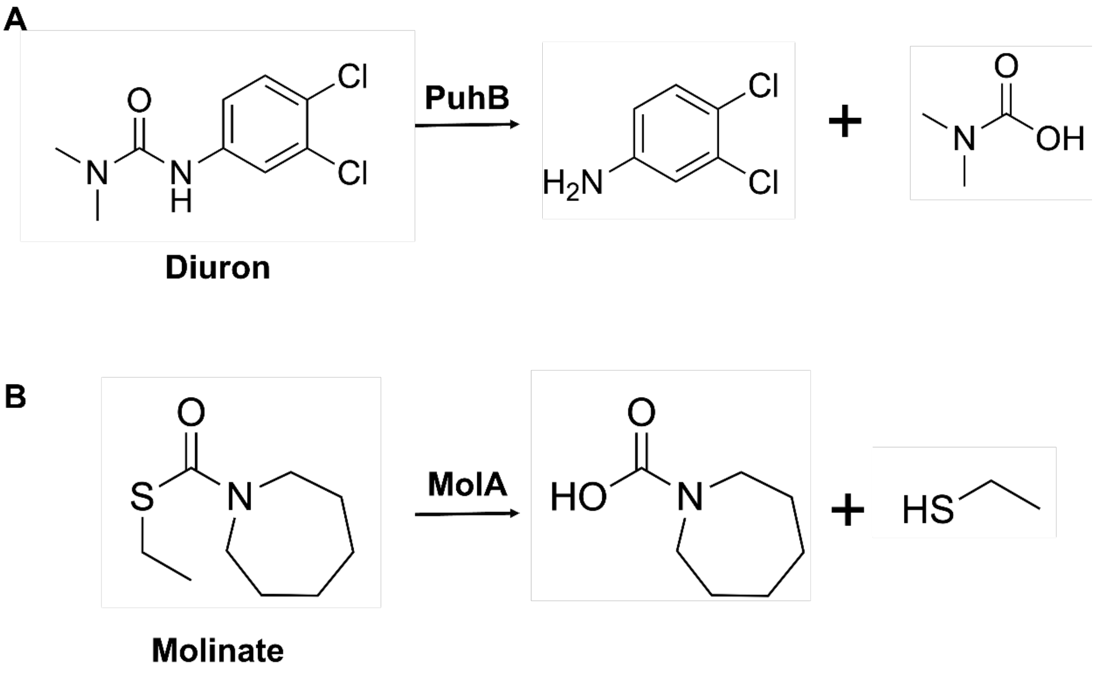

Supplementary Fig. 29. Metabolic functions of PuhB and MolA.

A, PuhB and **B** MolA respectively hydrolyze C-N bond in diuron and C-S bond in molinate.

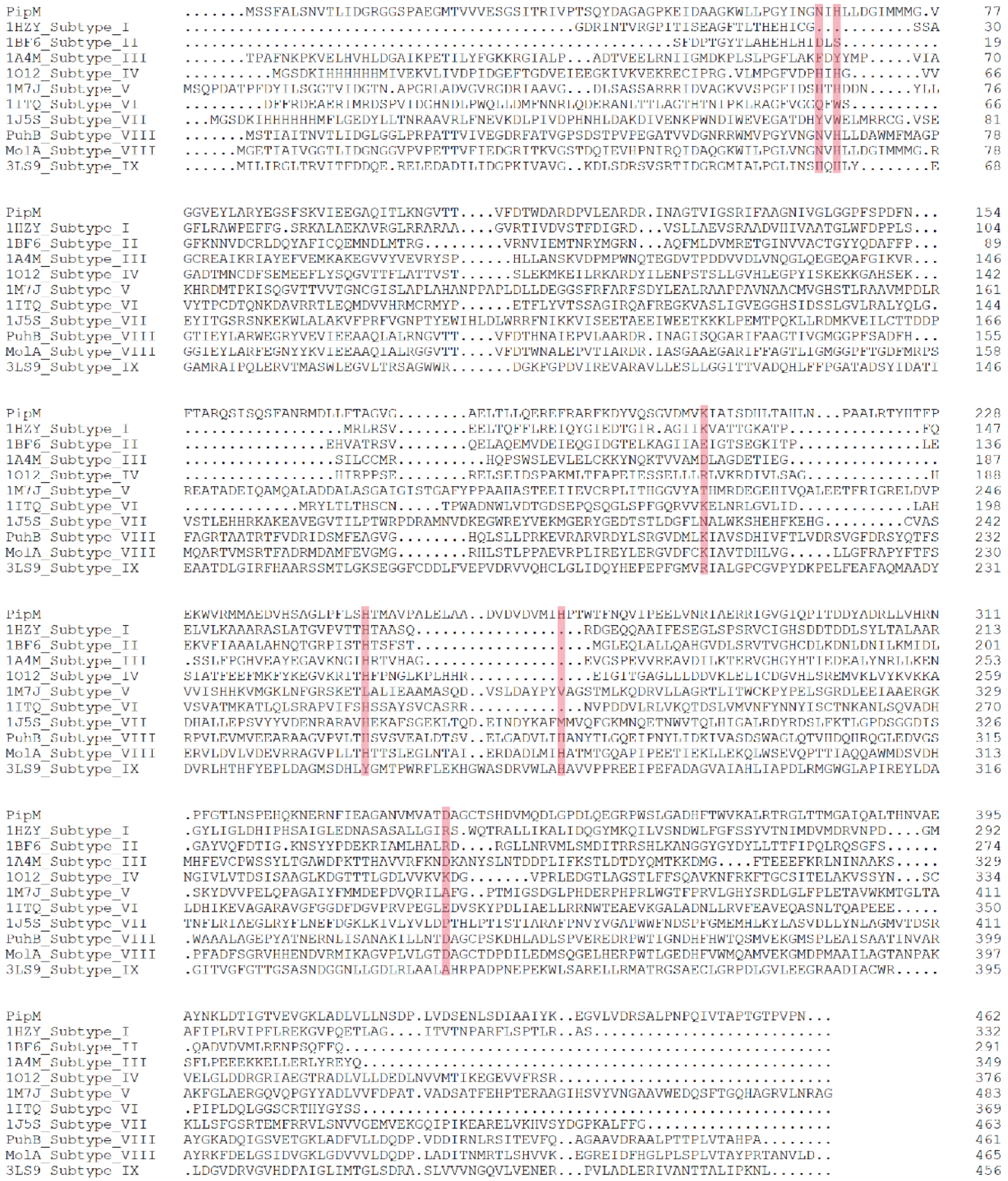

Supplementary Fig. 30. Multiple-sequence alignment of PipM with selected AHS enzymes.

Amino acid sequences of PipM and following AHS enzymes that are representative in each subtype: subtype I, PDB code 1HZY; subtype II, PDB code 1BF6; subtype III, PDB code 1A4M; subtype IV, PDB code 1O12; subtype V, PDB code 1M7J; subtype VI, PDB code 1M7J; subtype VII, PDB code 1J5S; subtype VIII, PuhB (PDB code 4WHB) and MolA (PDB code 4WGX); subtype IX, PDB code 3LS9. Shaded red indicates essential amino acid residues of metal-binding site.

```
PipM    MSSFALSNVTLIDGRGGSPAEGMTVVVESGSITRIVPTSQYDAGAGPKEIDAAGKWLLPG     60
PuhB    MSTIAITNVTLIDGLGGLPRPATTVIVEGDRFATVGPSDSTPVPEGATVVDGNRRWMVPG     60

PipM    YINGNIHLLDGIMMMG.VGGVEYLARYEGSFSKVIEEGAQITLKNGVTTVFDTWDARDPV    119
PuhB    YVNGNVHLLDAWMFMAGPGTIEYLARWEGRYVEVIEEAAQLALRNGVTTVFDTHNAIEPV    120

PipM    LEARDRINAGTVIGSRIFAAGNIVGLGGPFSPDFNFTARQSISQSFANRMDLLFTAGVGA    179
PuhB    LAARDRINAGISQGARIFAAGTIVGMGGPFSADFHFAGRTAATRTFVDRIDSMFEAGVGH    180

PipM    ELTLLQEREFRARFKDYVQSGVDMVKIAISDHLTAHLNPAAL...RTYHTFPEKWVRMMA    236
PuhB    QLSLLPRKEVRARVRDYLSRGVDMLKIAVSDHIVFTLVDRSVGFDRSYQTFSRPVLEVMV    240

PipM    EDVHSAGLPFLSHTMAVPALELAADVDVDVMIHPTWTFNQVIPEELVNRIAERRIGVGIQ    296
PuhB    EEARAAGVPVLTHSVSVEALDTSVELGADVLIHANYTLGQEIPNYLIDKIVASDSWAGLQ    300

PipM    PITDDYADRLLVHRNPFGTLNSPEHQKNERNFIEAGANVMVATDAGCTSHDVMQDLGPDL    356
PuhB    TVHDQHRQGLEDVGSWAAALAGEPYATNERNLISANAKILLNTDAGCPSKDHLADLSPVE    360

PipM    QEGRPWSLGADHFTWVKALRTRGLTTMGAIQALTHNVAEAYNKLDTIGTVEVGKLADLVL    416
PuhB    REDRPWTIGNDHFHWTQSMVEKGMSPLEAISAATINVARAYGKADQIGSVETGKLADFVL    420

PipM    LNSDPLVDSENLSDIAAIYKEGVLVDRSALPNPQIVTAPTGTPVP                   461
PuhB    LDQDPVDDIRNLRSITEVFQAGAAVDRAALPTTPLVTAHPA....                   461
```

Supplementary Fig. 31. Amino acid sequence alignment between PipM and PuhB.

Orange highlight: amino acid residues that formed lid-like structure.

Supplementary Tables

Supplementary Table 1. Bacterial strains, plasmids and primers.

**Strains**	
E. coli DH10B	Strain for cloning; F– *mcrA* Δ(*mrr-hsdRMS-mcrBC*) φ80*lacZ*ΔM15 Δ*lacX74 recA1 endA1 araD139* Δ (*ara-leu*)7697 *galU galK* λ– *rpsL*(Str^R) *nupG*
E. coli Rosetta™ 2	Strain for expression; F^-*ompT hsdS*$_B$(r_B^- m_B^-) *gal dcm* (DE3) pRARE2 (Cam^R)
**Plasmids**	
pET24a(+)	T7 RNA polymerase-dependent recombinant protein expression vector, Kan^R
pET24a(+)-*pipM*	*pipM* fragment (1389 bp) was inserted into the *Nde*I and *Hind*III sites of pET24a(+)
pET24a(+)-*pipUhis*	*pipU* fragment (1575 bp) was inserted into the *Nde*I and *Xho*I sites of pET24a(+)
pET24a(+)-*pipVhis*	*pipV* fragment (912 bp) was inserted into the *Nde*I and *Xho*I sites of pET24a(+)
pET24a(+)-*pipW*	*pipW* fragment (891 bp) was inserted into the *Nde*I and *Xho*I sites of pET24a(+)
pET24a(+)-*pipXhis*	*pipX* fragment (474 bp) was inserted into the *Nde*I and *Xho*I sites of pET24a(+)
**Primers**	
pipM Fw (*Nde*I)	TAAGAAAGGAGATATACATATGTCTAGTTTTGCGTTATCGAATGTCACGCTGATCG
pipM Rv (*Hind*III)	CTCGAGTGCGGCCGCAAGCTTTCAGTTCGGAACAGGCGTGCCC
pipU Fw (*Nde*I)	TAAGAAGGAGATATACATATGATCTATCGACCCCGAACCGTCGAACT
pipU Rv (*Hind*III)	CTCGAGTGCGGCCGCAAGCTTTGCGGATTCGACCACGTTCTGCTGC
pipV Fw (*Nde*I)	TAAGAAGGAGATATACATATGGATTTGAACAACAAGGTCGCAATCGTCA
pipV Rv (*Hind*III)	CTCGAGTGCGGCCGCAAGCTTTCGTGACATCAGCTCGTCCGGGAAC
pipW Fw (*Nde*I)	TAAGAAGGAGATATACATATGACGAAGTACGAGTACGGAATCGACTTCG
pipW Rv (*Xho*I)	GTGGTGGTGGTGGTGGTGCTCGAGTCATTCTGCGATACCTAGAACGCGAA
pipX Fw (*Nde*I)	TAAGAAGGAGATATACATATGACCACCTCCGCCCCTG
pipX Rv (*Hind*III)	CTCGAGTGCGGCCGCAAGCTTGTTGTAGTAGCGCAGAACTGCTTCGG

Supplementary Table 2. Piperine-metabolizing gene cluster.

Enzyme-coding genes related to piperine metabolism are marked in red. Numbers of ORFs are consistent with results shown in **Fig. 3**. Inf., one of two transcription levels was undetectable in this ORF, rendering accurate comparison impossible. ORF, open reading frame.

Gene	Putative activity of the deduce amino acid sequences	Transcription (Log2)
orf1	Hypothetical peptidase	3.68
orf2	Hypothetical protein	4.26
orf3	Aldehyde dehydrogenase	3.97
orf4	Gamma-glutamyl-gamma-aminobutyrate hydrolase	4.27
orf5	Amino acid ABC transporter substrate-binding protein	3.73
orf6	Glutamine synthetase	4.05
orf7	Ferredoxin reductase	5.32
orf8	Ferredoxin	3.98
orf9	Cytochrome P450	4.06
orf10	Amino acid permease	3.28
orf11	Hypothetical protein	2.74
orf12	GntR family transcriptional regulator	3.03
orf13	Hypothetical protein	4.70
orf14	Hypothetical protein	4.03
orf15	CoA transferase	4.15
orf16	Aldehyde dehydrogenase	4.48
orf17	Glutaryl-CoA dehydrogenase	4.57
orf18	Aspartate aminotransferase family protein	3.62
orf19	Putative NAD-glutamate dehydrogenase	2.35
orf20	Transposase	4.90
orf21	Hypothetical protein	4.69
orf22	Hypothetical protein	5.41
orf23	MFS transporter	5.69
orf24 (pipX)	MaoC family dehydratase	5.29
orf25 (pipW)	4-Hydroxyphenyl-beta-ketoacyl-CoA hydrolase	5.44
orf26 (pipV)	Dehydrogenase	5.77
orf27 (pipU)	Putative acyl-CoA synthetase	5.68
orf28	PaaX family transcriptional regulator	2.06
orf29	Putative fatty-acid-CoA ligase	4.49
orf30	Hypothetical protein	2.40
orf31	Hypothetical protein	0.722
orf32	Hypothetical protein	-0.169
orf33	Transposase	1.94
orf34	Hypothetical protein	1.36
orf35 (pipM)	Amidohydrolase	2.80
orf36	Hypothetical protein	3.25
orf37	Hypothetical protein	1.62
orf38	Hypothetical protein	2.33
orf39	DDE transposase	4.49
orf40	Dihydropteroate synthase	7.30
orf41	Bifunctional protein FolD	6.94
orf42	Hypothetical protein	Inf.
orf43	Hypothetical protein	2.19
orf44	Hypothetical protein	2.93
orf45	Hypothetical protein	3.57
orf46	Formyltetrahydrofolate deformylase	7.94
orf47 (pipA)	Glycine cleavage system protein T	8.64
orf48	MFS transporter	5.97

Supplementary Table 3. Metal analysis of PipM.

	PipM	Mg	Fe	Ni	Zn
Concentration (μM)	40	1.3	0.49	1.2	12.0

Supplementary Table 4. Comparison of amino acid sequences among PipU, V, W and X and homologues of *Rhodococcus jostii* RHA1 and *Agrobacterium fabrum* sp.

CouL, CouM, CouN and CouO are from *Rhodococcus jostii* RHA1. Atu1415, Atu1416, Atu1417, Atu1421 are from *Agrobacterium fabrum* sp.

Enzyme	Homologue (NCBI accession No.)	Coverage (%)	Similarity (%)	E value
PipU	CouL (WP_025431956)	92	50	5e-73
	Atu1416 (NP_354423)	54	37	2e-08
PipV	CouN (WP_005243844)	100	90	0
	Atu1415 (NP_354422)	65	49	1e-21
PipW	CouO (WP_011597356)	99	90	0
	Atu1421 (NP_354429)	95	69	3e-113
PipX	CouM (WP_005243848)	90	69	6e-55
	Atu1417 (NP_354425)	-	None	-

Supplementary Table 5. Comparison of amino acid sequences between PipU and acyl-CoA synthetase.

Enzyme	Original strain	NCBI accession No/ PDB code	Similarity (%) to PipU	E value
Long-chain-fatty-acid-CoA ligase FadD2	*Pseudomonas* sp.	WP_274120171	49	7e-78
Fatty-acid-CoA synthetase	*Mycobacterium tuberculosis* H37Rv	8R2Q	46	2e-38

Supplementary Table 6. Comparison of amino acid sequences between PipV and tri-functional enzyme (TFE) complex.

Enzyme	Original strain	PDB code	Similarity (%) to PipV
Fatty acid β-oxidation multienzyme complex	*Pseudomonas fragi*	1WDK	None
Aerobic trifunctional enzyme subunit-α	*Escherichia coli* K-12	6TNM	None
β-oxidation trifunctional enzyme α	*Mycobacterium tuberculosis* H37Rv	7O1I	None

Supplementary Table 7. Comparison of amino acid sequences between PipW and TFE complex.

Enzyme	Original strain	PDB code	Similarity (%) to PipW
Fatty acid β-oxidation multienzyme complex	*Pseudomonas fragi*	1WDK	None
Aerobic trifunctional enzyme subunit-α	*Escherichia coli* K-12	6TNM	None
β-oxidation trifunctional enzymeα	*Mycobacterium tuberculosis* H37Rv	7O1I	None

Supplementary Table 8. Comparison of amino acid sequences between PipX and TFE complex.

Enzyme	Original strain	PDB code	Similarity (%) to PipX
Fatty acid β-oxidation multienzyme complex	*Pseudomonas fragi*	1WDK	None
Aerobic trifunctional enzyme subunit-α	*Escherichia coli* K-12	6TNM	None
β-oxidation trifunctional enzyme α	*Mycobacterium tuberculosis* H37Rv	7O1I	None
